# Protein arginine methyltransferase PRMT1 promotes adipogenesis by modulating transcription factors C/EBPβ and PPARγ

**DOI:** 10.1016/j.jbc.2022.102309

**Published:** 2022-07-31

**Authors:** Qi Zhu, Dinghui Wang, Feng Liang, Xian Tong, Ziyun Liang, Xiaoyu Wang, Yaosheng Chen, Delin Mo

**Affiliations:** State Key Laboratory of Biocontrol, School of Life Sciences, Sun Yat-sen University, Guangzhou, Guangdong, China

**Keywords:** adipogenesis, PRMT1, PPARγ, Wnt/β-catenin, C/EBPβ, CCK-8, cell-counting kit 8, co-IP, coimmunoprecipitation, DAPI, 4′,6-diamidino-2-phenylindole, FBS, fetal bovine serum, GO, gene ontology, IP, immunoprecipitation, MCE, mitotic clonal expansion, ORO, oil red O staining, qPCR, quantitative PCR, WAT, white adipose tissue

## Abstract

Protein arginine methyltransferase 1 (PRMT1) methylates a variety of histone and nonhistone protein substrates to regulate multiple cellular functions such as transcription, DNA damage response, and signal transduction. It has been reported as an emerging regulator of various metabolic pathways including glucose metabolism in the liver, atrophy in the skeletal muscle, and lipid catabolism in the adipose tissue. However, the underlying mechanisms governing how PRMT1 regulates adipogenesis remain elusive. Here, we delineate the roles of PRMT1 in mitotic clonal expansion and adipocyte differentiation. Gain and loss of functions demonstrate that PRMT1 is essential for adipogenesis of 3T3-L1 and C3H10T1/2 cells. Mechanistically, we show PRMT1 promotes the expression of transcription factor peroxisome proliferator-activated receptor-γ (PPARγ) by catalyzing histone modification H4R3me2a and impedes the activation of Wnt/β-catenin signaling by increasing the level of Axin to accelerate adipogenic differentiation. In addition, we demonstrate mitotic clonal expansion is suppressed by PRMT1 deficiency. PRMT1 interacts with transcription factor CCATT enhancer-binding protein β (C/EBPβ), and the absence of PRMT1 leads to the depressed phosphorylation of C/EBPβ. Interestingly, we discover PRMT1 acts as a positive regulator of C/EBPβ protein stability through decreasing the level of E3 ubiquitin ligase Smurf2, which promotes the ubiquitination and degradation of C/EBPβ, thus facilitating adipogenesis. Collectively, these discoveries highlight a critical role of PRMT1 in adipogenesis and provide potential therapeutic targets for the treatment of obesity.

Obesity is an abnormal lipid metabolism and resulted from excessive expansion of white adipose tissue (WAT), closely related to multiple metabolism diseases, such as type 2 diabetes, atherosclerosis, coronary artery disease, adiposis hepatica, and hypertension (1, 2). WAT plays a significant role in energy homeostasis, storing triglycerides when energy is excess and releasing free fatty acids in the demand of energy. Besides, WAT serves as a crucial regulator of whole body metabolism by synthesizing and secreting adipokines, such as leptin and adiponectin (3). Adipose mass is dependent on adipocyte hypertrophy and adipocyte hyperplasia, defined as hypertrophic WAT and hyperplastic WAT, respectively (4). Adipocyte hypertrophy is an increase in adipocyte size, while adipocyte hyperplasia is a vital process that generates new adipocytes from precursor cells to guarantee the number of adipocytes (5). Consequently, understanding the mechanisms regulating both the number and size of adipocytes may provide new therapies to neutralize obesity and obesity-associated metabolic diseases.

Adipocytes are originated from pluripotent mesenchymal stem cells, which are committed into adipocyte lineage when appropriately stimulated (6). Then preadipocytes undergo a complicated process including growth arrest, mitotic clonal expansion (MCE), and terminal differentiation. Growth-arrested preadipocytes enter the cell cycle again and undertake cell divisions in the early stage of adipocyte differentiation, known as MCE, in which transcriptional factor CCATT enhancer-binding protein β (C/EBPβ) is of critical importance that activates cell cycle genes (7, 8). C/EBPβ is phosphorylated and activated by MAP kinase and GSK3β to trigger the transcription of C/EBPα and peroxisome proliferator–activated receptor-γ (PPARγ), which in turn coordinately induce the transcription of adipogenic genes to promote the formation of mature adipocytes (6). PPARγ is indispensable for adipogenic programming, considered the master regulator of adipogenesis. In particular, the adipogenesis of precursor cells is prevented in the absence of PPARγ, that cannot be restored by C/EBPα (9). Moreover, adipose tissue-specific PPARγ deletion of mice hinders high fat diet–induced obesity (10). Therefore, understanding the regulators that affect the expression and activity of PPARγ and C/EBPβ is of great significance to delve deeper into the mechanisms orchestrating adipogenesis.

Arginine methylation is perceived as a pivotal posttranslational modification controlling transcription, translation, protein stability, cell fate determination, and so forth (11). Arginine methylation is mainly catalyzed by PRMT family, subdivided into three categories according to their catalytic activity: type I enzymes (PRMT1–4, 6, and 8), which carry out the formation of asymmetrically dimethylated arginine, type II (PRMT5 and 9), and type III (PRMT7) enzymes, which catalyze symmetrical dimethylation and monomethylation, respectively (12). Growing studies have shown that PRMTs function as activators or repressors in many biological processes including adipogenesis (13, 14, 15, 16, 17). Arginine dimethylation of C/EBPβ catalyzed by PRMT4/CARM1 constrains the interaction among C/EBPβ with SWI/SNF and mediator complexes, and compromises its transactivation, that can be rescued by phosphorylation of C/EBPβ (13). Besides, PRMT5 has been found to regulate fatty acid metabolism and lipid droplet biogenesis by methylating SPT5 or SREBP1a and promote adipogenesis *via* activation of PPARγ and its target genes (14, 15). In contrast, PRMT6 has been identified to suppress adipogenic differentiation by repressing the activity of PPARγ (16). Additionally, PRMT7 has been shown to inhibit adipogenesis through modulating arginine methylation of C/EBPβ (17). However, the functions of other PRMTs in adipogenesis are still unclear. What is strikingly noticeable is PRMT1 regulating thermogenic fat activation (18). Moreover, recent study has reported that adipocyte-specific depletion of PRMT1 impairs glucose homeostasis in diet-induced obesity (19), but the regulatory mechanism of PRMT1 in adipogenesis requires further elucidation.

In this research, we find PRMT1 depletion represses adipogenesis in 3T3-L1 cells and C3H10T1/2 cells and attenuates adipogenic genes expression, whereas PRMT1 overexpression has the opposite effects. Gene ontology (GO) analysis reveals that PRMT1 participates in multifarious biological processes such as cell proliferation, fat cell differentiation, and lipid metabolic process. Furthermore, we demonstrate PRMT1 regulates adipocyte differentiation by mediating H4R3me2a at PPARγ promoter and impeding the activation of Wnt/β-catenin signaling by regulating Axin. Meanwhile, PRMT1 is also required for MCE. PRMT1 interacts with C/EBPβ, and the absence of PRMT1 leads to the depressed phosphorylation of C/EBPβ. We further identify PRMT1 as a positive regulator of C/EBPβ protein stability through decreasing the level of Smad ubiquitination regulatory factor 2 (Smurf2), which promotes the ubiquitination and degradation of C/EBPβ, thus facilitating adipogenesis. Overall, our findings elucidate that PRMT1 is a crucial and multifaceted regulatory factor that facilitates adipogenesis at both the transcriptional and posttranslational level.

## Results

### PRMT1 promotes adipogenic differentiation in 3T3-L1 cells

**Figure 1:**
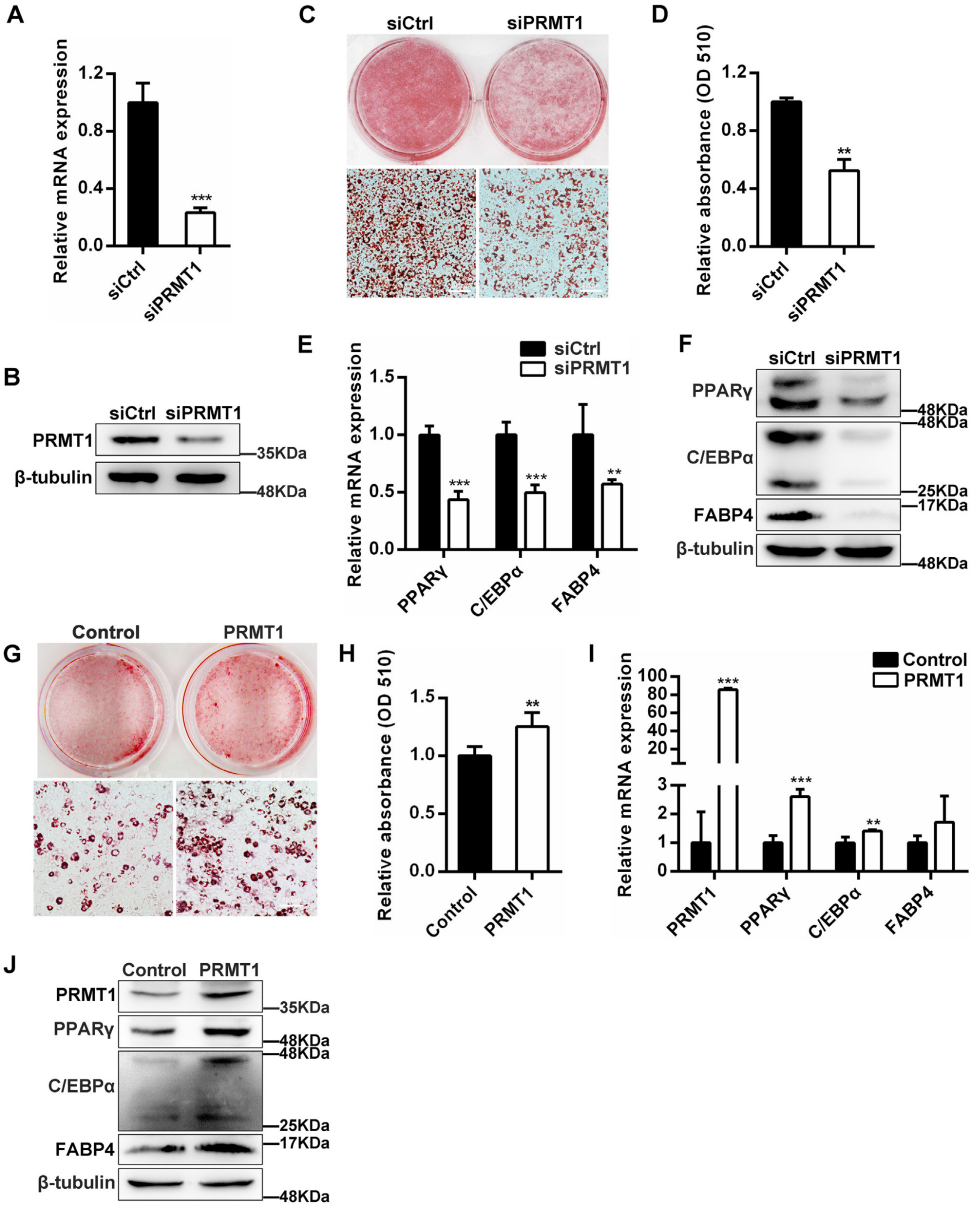
**Depletion of PRMT1 inhibits the differentiation of preadipocytes.***A*, PRMT1 mRNA expression in siCtrl and siPRMT1 3T3-L1 cells. *B*, Western blot determined the efficiency of PRMT1 silence in 3T3-L1 cells. *C*, effect of PRMT1 knockdown or negative control on 3T3-L1 adipogenic differentiation assessed by oil red O staining. The scale bar represents 200 μm. *D*, the absorbance values were measured at 510 nm as shown in (*C*). Results are the mean ± SD (n = 3). Student’s *t* test, ∗∗*p* < 0.01. *E*, the mRNA levels of genes relevant to adipogenesis in siCtrl or siPRMT1 3T3-L1 cells at 6 days. Data are represented as mean ± SD (n = 3). Student’s *t* test, ∗∗*p* < 0.01; ∗∗∗*p* < 0.001. *F*, Western blot analysis for adipogenic marker genes expression from differentiated 3T3-L1 adipocytes. *G*, oil red O staining of adipocytes overexpressed with control or PRMT1 plasmids at day 6. The scale bar represents 200 μm. *H*, the absorbance value of ORO extracted from plates stained as shown in (*G*) was measured at *A*_510_ nm. Data are mean ± SD (n = 3). Student’s *t* test, ∗∗*p* < 0.01. *I*, adipogenic marker genes mRNA expression in siCtrl and siPRMT1 3T3-L1 cells post-differentiation. Data represent mean ± SD (n = 3). Student’s *t* test, ∗∗*p* < 0.01, ∗∗∗*p* < 0.01. *J*, Western blot of the indicated genes from differentiated siCtrl and siPRMT1 3T3-L1 cells corresponding to I.

To explore the specific role of PRMT1 in adipogenesis, the expression of PRMT1 was knocked down in 3T3-L1 preadipocytes using siRNA transfection (Fig. S1). The mRNA and protein levels of PRMT1 were detected to authenticate the efficiency of siRNA interference (Fig. 1, *A* and *B*). As a result, PRMT1 knockdown suppressed adipogenic differentiation of 3T3-L1 cells, as determined by oil red O staining (ORO) (Fig. 1, *C* and *D*). In addition, the expression of genes related to adipogenesis was monitored by quantitative RT-PCR and Western blot. A significant decrease was observed in the expression of C/EBPα, PPARγ, and FABP4, serving as markers of adipogenesis, upon PRMT1 depletion (Fig. 1, *E* and *F*). Moreover, the effect of PRMT1 overexpression on 3T3-L1 adipocyte differentiation was evaluated using plasmids transfection. The results of ORO indicated that overexpression of PRMT1 significantly increased lipid droplets formation (Fig. 1, *G* and *H*). Consistently, the expression of C/EBPα, PPARγ, and FABP4 at both mRNA (Fig. 1*I*) and protein (Fig. 1*J*) levels were increased when PRMT1 was overexpressed in 3T3-L1 cells. These results prove that PRMT1 is required for the normal adipocyte differentiation.

### PRMT1 accelerates adipogenesis of C3H10T1/2 cells

**Figure 2:**
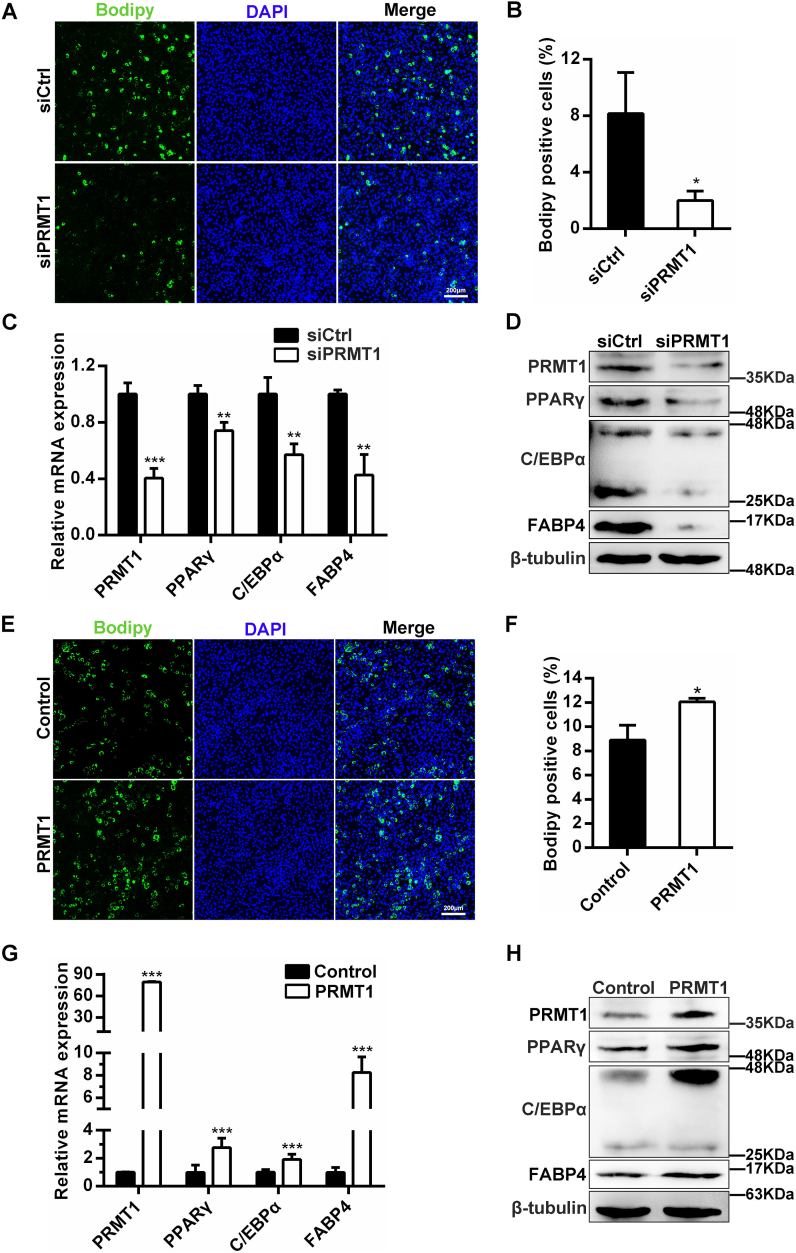
**PRMT1 is required for adipogenesis of C3H10T1/2 mesenchymal stem cells.***A*, C3H10T1/2 cells transfected with siCtrl or siPRMT1 were induced for adipogenic differentiation. Lipid accumulation was estimated by Bodipy staining. The scale bar represents 200 μm. *B*, the percentage of Bodipy positive cells was counted as shown in (*A*). Results are the mean ± SD (n = 3). Student’s *t* test, ∗*p* < 0.05. *C*, relative mRNA expression by qRT-PCR of indicated genes in differentiated siCtrl or siPRMT1 C3H10T1/2 cells at day 6. *D*, Western blot showing the levels of C/EBPα, PPARγ and FABP4 after knockdown PRMT1 in C3H10T1/2 cells at day 6. *E*, Bodipy staining of differentiated C3H10T1/2 cells expressing empty vector (control) or PRMT1 at day 6. The scale bar represents 200 μm. *F*, statistical analysis of Bodipy staining in (*E*). Results are the mean ± SD (n = 3). Student’s *t* test, ∗*p* < 0.05. *G*, qRT-PCR was performed to determine the mRNA levels of adipogenic transcription factors in siCtrl or siPRMT1 C3H10T1/2 cells at day 6. Data are shown as mean ± SD (n = 3). Student’s *t* test, ∗∗∗*p* < 0.001. *H*, Western blot analysis of the expression of adipogenesis markers. qRT-PCR, quantitative RT-PCR.

C3H10T1/2 cells were employed to probe into the effect of PRMT1 on adipogenesis of mesenchymal stem cells that can commit and differentiate into multiple lineages depending upon the signaling pathways activated by different differentiation cocktails. C3H10T1/2 cells were treated with BMP4 to induce the adipogenic commitment. Then, DMI, a cocktail of differentiation agents containing dexamethasone, 3-isobutyl-1-methylxanthine, and insulin, was employed to induce the adipogenesis of C3H10T1/2 cells. When PRMT1 levels were reduced by siRNA in C3H10T1/2 cells, upon adipogenesis, we observed a significant decrease in both lipid accumulation (based on Bodipy staining) (Fig. 2, *A* and *B*) and adipogenic marker genes expression (Fig. 2, *C* and *D*). On the contrary, Bodipy staining data indicated that transient overexpression of PRMT1 facilitated adipogenic differentiation of C3H10T1/2 cells (Fig. 2, *E* and *F*). Furthermore, the mRNA and protein expression levels of the adipogenic marker genes C/EBPα, PPARγ, and FABP4 were significantly higher in PRMT1 overexpression cells compared with the control (Fig. 2, *G* and *H*). Collectively, our results strongly suggest that PRMT1 positively regulates the adipogenesis of mesenchymal stem cells.

### PRMT1 accelerates adipogenic differentiation by blocking the activation of Wnt/β-catenin signaling

**Figure 3:**
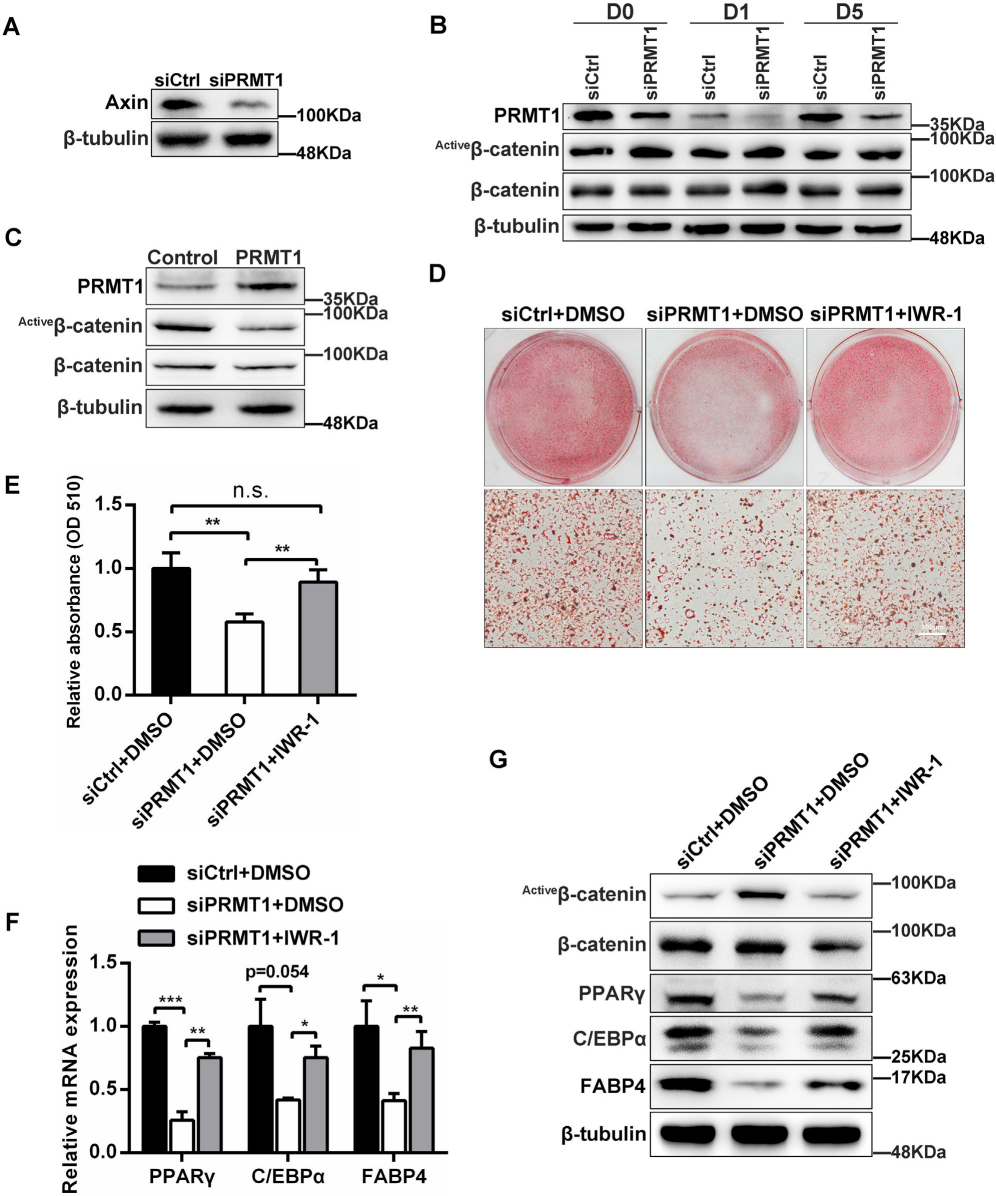
**PRMT1 deficiency promotes the activation of Wnt/β-catenin signaling pathway in 3T3-L1 cells.***A*, Western blot analysis of the Axin protein expression. *B*, protein levels of PRMT1, β-catenin (total), and active β-catenin at day 0, 1, and 5 were detected by Western blot when PRMT1 was silenced in 3T3-L1 cells. *C*, Western blot showing the relative levels of PRMT1, β-catenin (total), and active β-catenin at day 5 in differentiated 3T3-L1 cells with or without overexpression of PRMT1. β-Tubulin was used as a loading control. *D*, 3T3-L1 cells were transfected with siCtrl or siPRMT1 and treated with DMSO or IWR-1, respectively. Oil red O staining was performed after 6 days of differentiation. The scale bar represents 200 μm. *E*, relative absorbance was calculated at 510 nm as shown in (*D*). Values are expressed as mean ± SD (n = 3). Student’s *t* test, n.s. not significance, ∗∗*p* < 0.01. *F*, the mRNA expression of C/EBPα, PPARγ, and FABP4 was detected by qRT-PCR at day 6 in 3T3-L1 cells transfected with siCtrl or siPRMT1 and treated with DMSO or IWR-1, respectively. Data are presented as mean ± SD (n = 3). Student’s *t* test, ∗*p* < 0.05, ∗∗*p* < 0.01, ∗∗∗*p* < 0.001. *G*, Western blot analysis of C/EBPα, PPARγ, FABP4, β-catenin (total), and active β-catenin at day 6 in 3T3-L1 cells transfected with siCtrl or siPRMT1 and treated with DMSO or IWR-1, respectively. DMSO, dimethyl sulfoxide; qRT-PCR, quantitative RT-PCR.

Previous study has shown that PRMT1 methylates Axin, a negative regulator of Wnt signaling, and enhances its stability to regulate Wnt/β-catenin signaling (20). Thus, we examined whether PRMT1 regulates adipocyte differentiation through Wnt/β-catenin signaling. In our study, the absence of PRMT1 resulted in the lessened level of Axin in 3T3-L1 cells (Fig. 3*A*). Moreover, knockdown of PRMT1 in 3T3-L1 cells enhanced the level of active β-catenin (nonphospho β-catenin Ser33/37/Thr41) (Fig. 3*B*). Conversely, the level of active β-catenin was remarkably decreased in the 3T3-L1 differentiated adipocytes with overexpression of PRMT1 compared to control (Fig. 3*C*). To further verify our hypothesis, we employed the IWR-1 endo, a specific β-catenin inhibitor that stabilizes Axin to block Wnt signaling pathway, to treat 3T3-L1 cells. As expected, IWR-1 restrained the incremental protein level of active β-catenin (Fig. 3*G*) and partially rescued the inhibition of lipid accumulation triggered by PRMT1 knockdown (Fig. 3, *D* and *E*). Meanwhile, the expression of adipogenic genes C/EBPα, PPARγ, and FABP4 were restored in the siPRMT1 + IWR-1 treatment group compared with the siPRMT1 + dimethyl sulfoxide group (Fig. 3, *F* and *G*). In summary, PRMT1 deficiency suppresses adipocyte differentiation *via* reducing the level of Axin and promoting the activation of Wnt/β-catenin signaling.

### PRMT1 promotes adipogenesis by mediating H4R3me2a methylation at the PPARγ promoter

**Figure 4:**
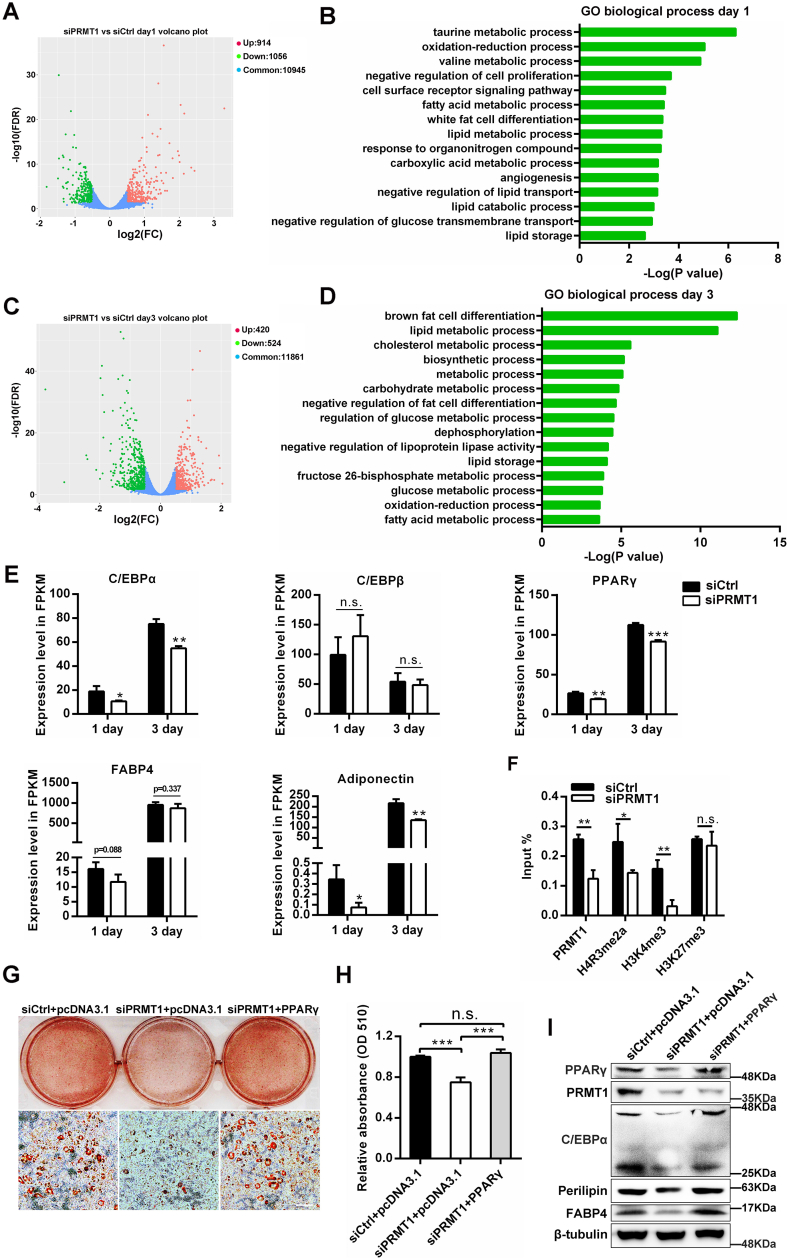
**PRMT1 promotes adipogenic differentiation through mediating H4R3me2a of PPARγ promoter.***A*, volcano plot of the differentially expressed genes between siPRMT1 and siCtrl 3T3-L1 cells at DMI induction for 1 day. *B*, gene ontology (GO) analysis of downregulated genes at day 1. The *p* value of biological process GO-term is shown. *C*, volcano plot of the differentially expressed genes between siPRMT1 and siCtrl 3T3-L1 cells at DMI induction for 3 days. *D*, GO analysis of differentially expressed genes at day 3 involved in lipid metabolism and fat cell differentiation. *E*, the expression levels in FPKM (fragments per kilobase of transcript per million) of master adipogenesis regulators in siPRMT1 and siCtrl 3T3-L1 cells at day 1 and day 3. Data are presented as mean ± SD. n.s. not significance, ∗*p* < 0.05, ∗∗*p* < 0.01, ∗∗∗*p* < 0.001. *F*, the enrichment of PRMT1, H4R3me2a, H3K4me3, and H3K27me3 by ChIP analysis at PPARγ promoter in siCtrl and siPRMT1 3T3-L1 cells. Data are presented as mean ± SD (n = 3). Student’s *t* test, n.s. not significance, ∗*p* < 0.05, ∗∗*p* < 0.01. *G*, the adipogenic phenotypes of 3T3-L1 cells transfected with siPRMT1 or siCtrl and empty or PPARγ vector after DMI induction for 6 days were assessed by oil red O staining. The scale bar represents 200 μm. *H*, colorimetric quantitation of oil red O staining shown in (*G*). Student’s *t* test, n.s. not significance, ∗∗∗*p* < 0.001. *I*, protein levels of PRMT1, PPARγ, Perilipin, C/EBPα, and FABP4 at day 6 were detected by Western blot. ChIP, chromatin immunoprecipitation.

To obtain a basic overview of the impact of PRMT1 on adipogenesis and explore the potential target genes, RNA sequencing was performed from control and PRMT1 knockdown 3T3-L1 cells with DMI treatment for 1 day (MCE stage) and 3 days (differentiation stage). Then, 914 upregulated genes and 1056 downregulated genes were identified for 1 day (Fig. 4*A*), while there were 420 upregulated genes and 524 downregulated genes for 3 days (Fig. 4*C*). By GO analysis, the downregulated genes were found to be mostly related to cell proliferation, white fat cell differentiation, and lipid metabolic progress at day 1 (Fig. 4*B*) and fat cell differentiation, lipid metabolic progress, and lipid storage at day 3 (Fig. 4*D*), indicating that PRMT1 is involved in proper regulation of fat cell differentiation and lipid metabolism. On the other hand, the upregulated transcripts were mainly related to defense response to virus, neuron differentiation, and cell proliferation at day 1 (Fig. S2*A*) and cell adhesion, signaling receptor activity, angiogenesis, and lipid transport at day 3 (Fig. S2*B*). The expression levels of pivotal adipogenesis regulator C/EBPα, C/EBPβ, PPARγ, FABP4, and adiponectin in fragments per kilobase of transcript per million mapped reads were shown in Figure 4*E*. Particularly, we found that knockdown of PRMT1 decreased the expression of PPARγ (Fig. 4*E*), a master regulator of adipogenesis and insulin responsiveness. Moreover, Wnt/β-catenin signaling has been demonstrated to suppress adipogenesis through reducing the expression of PPARγ (21). On the basis of our findings that PRMT1 promotes the expression of PPARγ in 3T3-L1 or C3H10T1/2 cells (Figs. 1, *E*, *F*, 2, *C* and *D*), we further sought to determine whether PRMT1 affects adipocyte differentiation through regulating PPARγ. Considering that PRMT1 is a predominant arginine methyltransferase, by chromatin immunoprecipitation analysis, we observed a significant decrease of PRMT1 and H4R3me2a recruitment to the PPARγ promoter in PRMT1 knockdown cells compared to control (Fig. 4*F*). Meanwhile, the enrichment of H3K4me3 at PPARγ promoter also diminished, while the binding of H3K27me3 to PPARγ promoter (Fig. 4*F*) and H4R3me2a to PPARγ enhancer-like region were unchanged (Fig. S2*C*). In addition, we found PRMT1 could also bind to the promoter of FABP4 (Fig. S2*D*). Although PRMT1 was hardly enriched to C/EBPα promoter, it cooperated with PPARγ to strength the transactivation of C/EBPα (Fig. S2, *D* and *E*). H4R3me2a, as an active histone modification mainly mediated by PRMT1, potentiates subsequent histone acetylation and contributes to establishing euchromatin structure (22, 23). Coincidentally, previous study reported that PRMT1 regulates macrophage differentiation *via* mediating H4R3me2a methylation at PPARγ promoter (24). To decipher whether PPARγ is a downstream-regulated gene of PRMT1 mediating the effects of adipogenic differentiation, 3T3-L1 cells were cotransfected with PRMT1 siRNA and PPARγ plasmid. Notably, Figure 4, *G* and *H* showed that overexpression of PPARγ could rescue the inhibited adipocyte differentiation caused by PRMT1 knockdown. Consistent with this, the expression of adipogenic genes including PPARγ and its target genes C/EBPα and FABP4 were markedly restored in cotransfection with siPRMT1 and PPARγ plasmid group (Fig. 4*I*). Together, these experiments confirm that PRMT1 regulates adipocyte differentiation *via* modulating H4R3me2a of PPARγ promoter.

### PRMT1 facilitates adipocyte differentiation dependent on its methyltransferase activity

**Figure 5:**
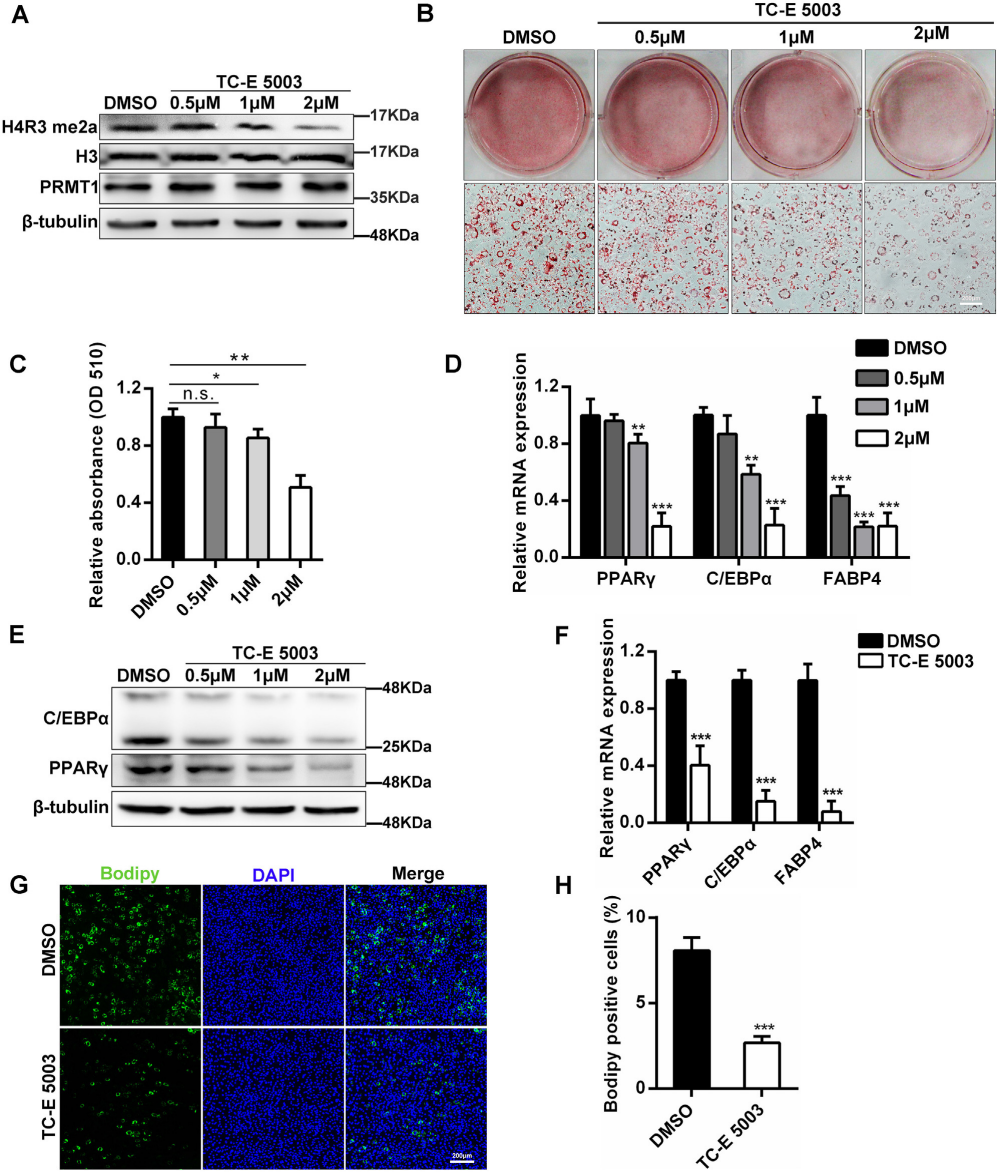
**The methyltransferase activity of PRMT1 is essential for adipocyte differentiation.***A*, 3T3-L1 cells were treated with vehicle (DMSO) or 0.5, 1, and 2 μM TC-E5003 for 36 h and then subjected to Western blot analyses using the corresponding antibodies. *B*, oil red O (ORO) staining of differentiated 3T3-L1 cells treated with vehicle (DMSO) or 0.5, 1, and 2 μM TC-E5003. The scale bar represents 200 μm. *C*, absorption of the eluate was measured photometrically at 510 nm for ORO. Data are shown as mean ± SD (n = 3). Student’s *t* test, n.s. not significance, ∗*p* < 0.05, ∗∗*p* < 0.01. *D*, relative mRNA levels of adipogenic marker genes in differentiated 3T3-L1 cells with the treatment of vehicle (DMSO) or 0.5, 1, and 2 μM TC-E5003 at day 6. Results are shown as mean ± SD (n = 3). Student’s *t* test, ∗*p* < 0.05, ∗∗*p* < 0.01, ∗∗∗*p* < 0.001. *E*, the protein levels of C/EBPα and PPARγ as indicated in (*D*). β-tubulin serves as the internal reference. *F*, qPCR analysis of C/EBPα, PPARγ, and FABP4 in C3H10T1/2 cells with or without TC-E 5003 (2 μM) after induction of adipocyte differentiation for 6 days. Data are presented as mean ± SD (n = 3). Student’s *t* test, ∗∗∗*p* < 0.001. *G*, Bodipy staining determined the adipogenic phenotypes of C3H10T1/2 cells treated with DMSO or TC-E 5003 (2 μM) after DMI induction for 6 days. The scale bar represents 200 μm. *H*, the quantification of Bodipy positive cells. Presented as mean ± SD (n = 3). Student’s *t* test, ∗∗∗*p* < 0.001. DMSO, dimethyl sulfoxide; qPCR, quantitative PCR.

To further expound the enzymes role of PRMT1 in adipogenesis, the selective PRMT1 inhibitor TC-E 5003 was adopted to block PRMT1 arginine methyltransferase function during adipogenic differentiation. As expected, the expression of H4R3me2a was dramatically reduced, and meanwhile, the protein level of PRMT1 remained unchanged (Fig. 5*A*). In accordance with the inhibitory role of PRMT1 silencing in 3T3-L1 cells, striking reduction was observed in lipid accumulation especially in the 2 μM TC-E 5003 treatment group, as assessed by ORO (Fig. 5, *B* and *C*). Furthermore, TC-E 5003 downregulated the mRNA and protein levels of the adipogenic marker genes C/EBPα, PPARγ, and FABP4 in a dose-dependent manner (Fig. 5, *D* and *E*). We also examined the function of TC-E5003 to C3H10T1/2 cells during adipogenesis. Similarly, the expression of C/EBPα, PPARγ, and FABP4 significantly decreased in C3H10T1/2 cells treated with TC-E 5003 compared with the control at 6 days of differentiation (Fig. 5*F*). In addition, Bodipy staining showed that accumulated lipid droplets of C3H10T1/2 cells was restrained in the presence of PRMT1 inhibitor TC-E 5003 (Fig. 5, *G* and *H*). Taken together, PRMT1 promotes adipocyte differentiation dependent on its methyltransferase activity.

### PRMT1 deficiency impairs MCE during adipogenesis

**Figure 6:**
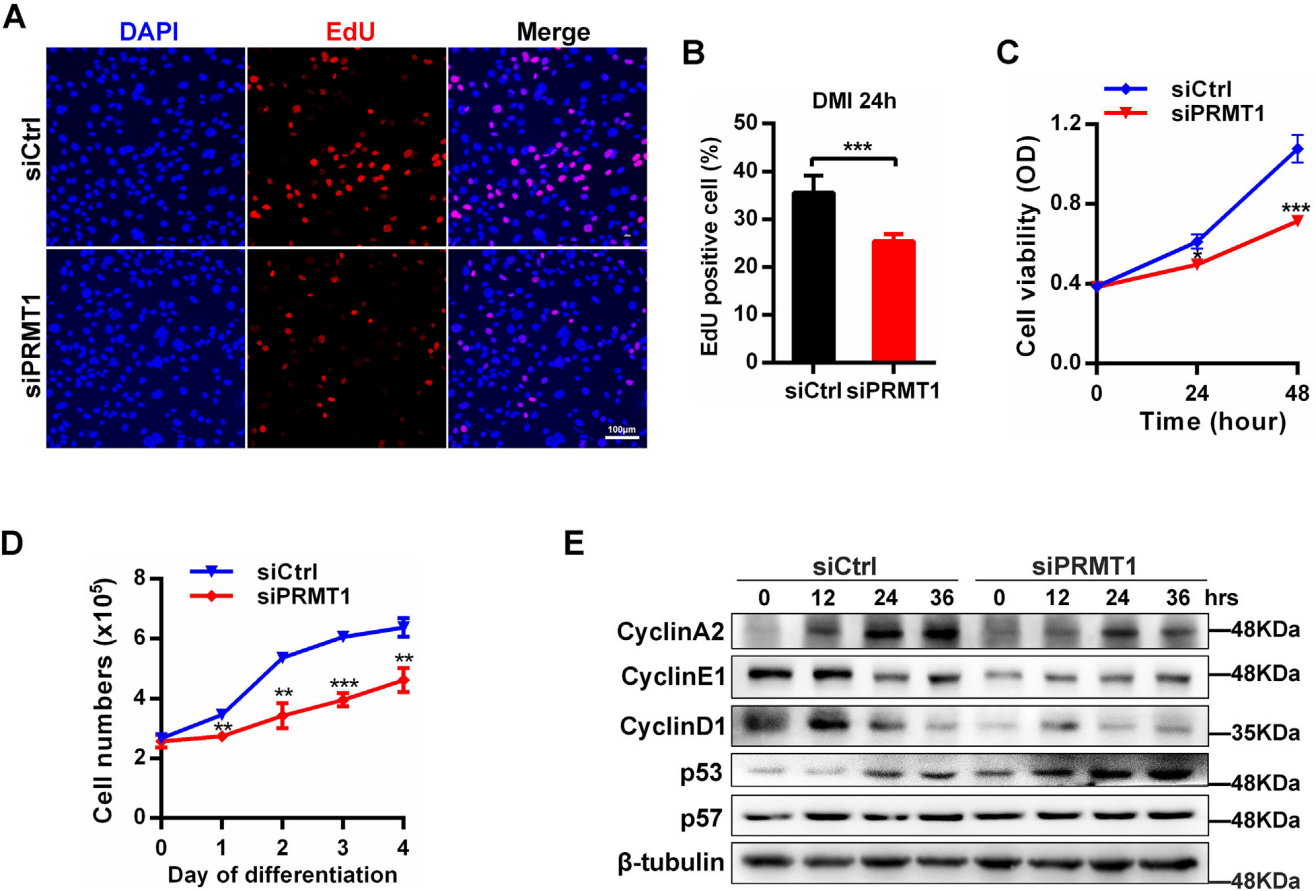
**PRMT1 deficiency hinders mitotic clonal expansion (MCE) during adipogenesis.***A*, representative images of the EdU staining for siCtrl or siPRMT1 3T3-L1 cells after DMI induction for 24 h. The scale bar represents 100 μm. *B*, the percentage of EdU-positive cells as shown in (*A*). *C*, 3T3-L1 cells transfected with siPRMT1 or siCtrl were subjected to the CCK-8 assay at DMI induction for 0, 24, and 48 h. Data are presented as mean ± SD (n = 3). ∗∗*p* < 0.01, ∗∗∗*p* < 0.001. *D*, 3T3-L1 cells transfected with siPRMT1 or siCtrl were induced for adipogenesis. Then, cells were counted at different time points. Data are presented as mean ± SD (n = 3). ∗*p* < 0.05, ∗∗∗*p* < 0.001. *E*, Western blot of cell cycle proteins in 3T3-L1 cells during MCE. CCK-8, cell counting kit 8.

Adipogenesis involves growth arrest, MCE, terminal differentiation, and adipocytes maturation, one of which occurs early is that growth arrest preadipocytes go through MCE (25). GO analysis at day 1, that is, in the stage of MCE, showed PRMT1 participated in cell proliferation (Fig. 4*B*). To investigate whether PRMT1 deficiency also affects MCE, we first utilized EdU incorporation assay to detect the proliferation of 3T3-L1 cells at DMI induction for 24 h (Fig. 6*A*), indicating that PRMT1 deficiency led to a decrease in proliferation (Fig. 6*B*). Similarly, cell counting kit-8 (CCK-8) assay proved that PRMT1 deletion significantly inhibited cell growth (Fig. 6*C*). We then counted the number of 3T3-L1 cells during MCE at different points (Fig. 6*D*). The growth curve suggested that PRMT1 depletion hindered MCE. To further explore this, the protein levels of cell cycle regulatory factors, such as cyclin A2, cyclin D1, cyclin E1, p53, and p57, were determined by Western blot. The expression level of cyclin A2, cyclin D1, and cyclin E1 was reduced, while there was no obvious change in the protein level of p57 (Fig. 6*E*). PRMT1 knockdown has been demonstrated to activate the p53 signal pathway and induce cell growth arrest in tumor (26). Likewise, we observed the significant increase of p53 when PRMT1 was silenced in 3T3-L1 cells (Fig. 6*E*). Additionally, quantitative RT-PCR demonstrated that loss of PRMT1 led to the upregulated expression of p53 target genes including p21, Sestrin2, and PGC1α (Fig. S3). Overall, these findings confirm that PRMT1 positively regulates MCE during adipogenesis.

Considering that PRMT1 affected MCE, to further evaluate whether PRMT1 may influence adipogenic differentiation independent of MCE, 3T3-L1 cells were transfected with siCtrl and siPRMT1 after DMI induction for 2 days when 3T3-L1 cells had gone through MCE. In accord with findings of Figure 1, Bodipy staining and ORO demonstrated PRMT1 deficiency suppressed terminal adipocyte differentiation (Fig. S4, *A*–*D*), accompanied by the lessened expression of adipogenic genes C/EBPα, PPARγ, Perilipin, adiponectin, and FABP4 (Fig. S4*E*). These findings reveal that PRMT1 orchestrates MCE and adipogenic differentiation, respectively.

### PRMT1 interacts with C/EBPβ and enhances the phosphorylation of C/EBPβ

**Figure 7:**
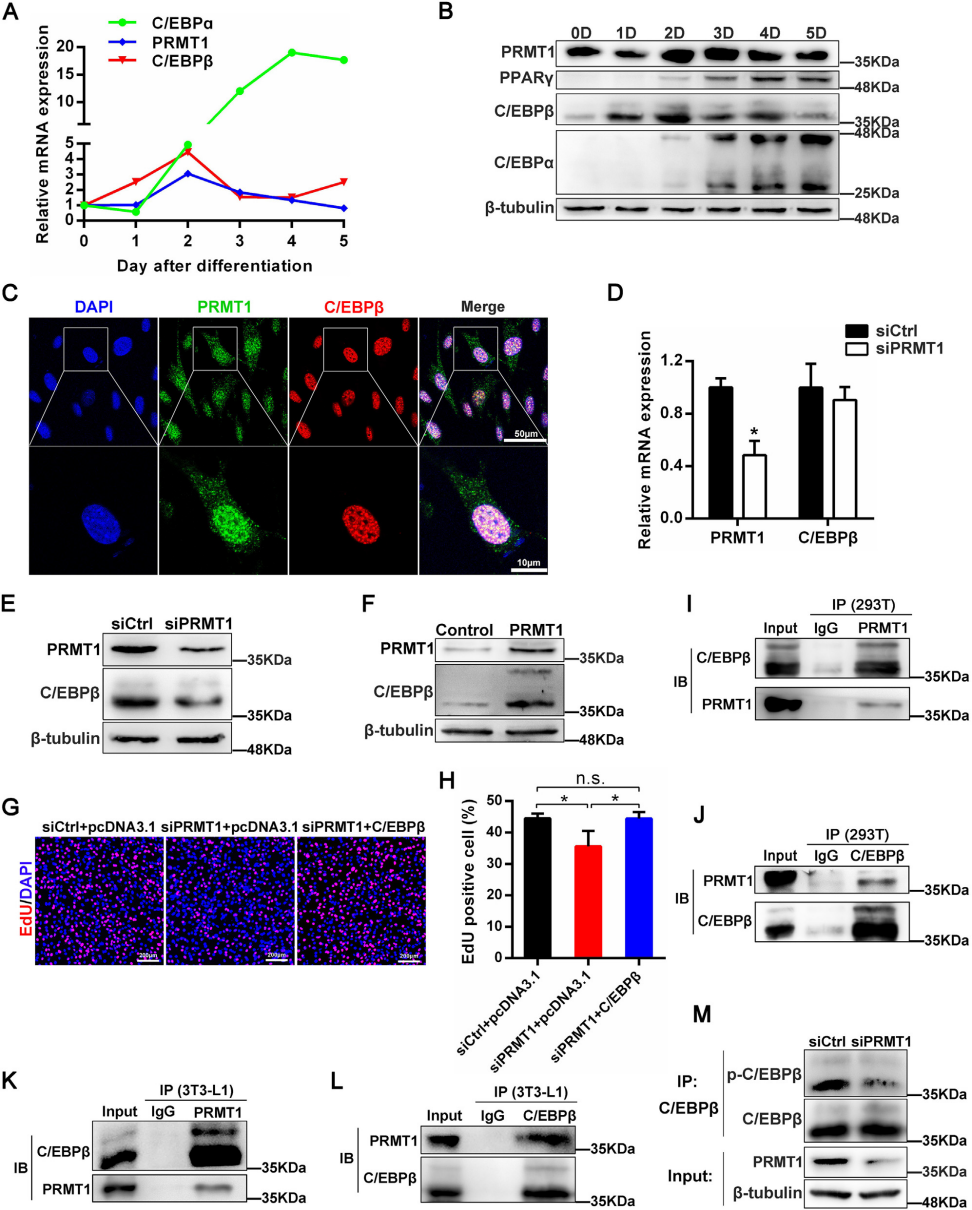
**PRMT1 interacts with C/EBPβ and reinforces the phosphorylation of C/EBPβ.***A*, 3T3-L1 preadipocytes were induced for adipogenesis. The mRNA expression levels of PRMT1, C/EBPɑ, and C/EBPβ were detected by qRT-PCR at the indicated time points. *B*, the protein profiles of PRMT1, C/EBPβ, C/EBPɑ, and PPARγ were determined by Western blot during adipocyte differentiation. *C*, representative confocal images displaying the colocalization between PRMT1 and C/EBPβ. *D*, mRNA expression of C/EBPβ in siCtrl and siPRMT1 3T3-L1 cells with the treatment of DMI for 2 days. Data are represented as mean ± SD (n = 3). ∗*p* < 0.05. *E*, protein expression of C/EBPβ in siCtrl and siPRMT1 3T3-L1 cells. *F*, protein level of C/EBPβ was detected in 3T3-L1 cells overexpressing control or PRMT1 plasmids with the treatment of DMI for 2 days. *G*, the effect of PRMT1 knockdown or together with overexpression of C/EBPβ on proliferation of 3T3-L1 cells at day 2. *H*, percentage of EdU-positive cells shown in (*G*). Data are represented as mean ± SD (n = 3). n.s. not significance, ∗*p* < 0.05. *I* and *J*, PRMT1 interacts with C/EBPβ in 293T cells. 293T cells were cotransfected with plasmids expressing PRMT1 and C/EBPβ. The interaction between PRMT1 and C/EBPβ was authenticated by immunoprecipitation using anti-PRMT1 antibody (*I*) or anti-C/EBPβ antibody (*J*). Immunoblotting was performed with the indicated antibodies. *K*, PRMT1 interacts with C/EBPβ in 3T3-L1 cells. 3T3-L1 cells with the treatment of DMI for 2 days were lysed for immunoprecipitation with anti-PRMT1 antibody and then analyzed by Western blot as indicated. *L*, C/EBPβ interacts with PRMT1 in 3T3-L1 cells. 3T3-L1 cells with the treatment of DMI for 2 days were lysed for immunoprecipitation with anti-C/EBPβ antibody and then analyzed by Western blot as indicated. *M*, 3T3-L1 cells transfected with siCtrl and siPRMT1 were induced with DMI for 2 days. Lyses were immunoprecipitated with anti-C/EBPβ antibody and then analyzed by Western blot as indicated. qRT-PCR, quantitative RT-PCR.

Previously, C/EBPβ was reported to upregulate mitosis-related genes expression, thereby facilitating MCE in the early progress of adipogenesis and triggering the transcription of PPARγ and C/EBPα in the late stage of differentiation (6). Interestingly, we detected the expression profiles of PRMT1 in adipogenesis similar to C/EBPβ (Fig. 7, *A* and *B*). By confocal microscopy in 3T3-L1 cells, we confirmed that PRMT1 colocalized in the nucleus with C/EBPβ (Fig. 7*C*). Although PRMT1 had little effect on the mRNA expression of C/EBPβ demonstrated by quantitative PCR (qPCR) (Fig. 7*D*) and RNA-seq data (Fig. 4*E*), the protein level of C/EBPβ was attenuated in PRMT1 deficiency 3T3-L1 cells (Fig. 7*E*). Conversely, overexpression of PRMT1 increased the protein level of C/EBPβ (Fig. 7*F*), whereas C/EBPβ in turn did not affect the protein expression of PRMT1 (Fig. S5). These data suggested that PRMT1 regulates C/EBPβ expression at posttranscriptional level. Furthermore, we found that C/EBPβ rescued the inhibited proliferation ability of MCE by PRMT1 knockdown (Fig. 7, *G* and *H*).

We next set out to explore the underlying regulatory mechanism between PRMT1 and C/EBPβ. PRMT1 and C/EBPβ plasmids were cotransfected into 293T cells and the interaction between PRMT1 and C/EBPβ were discovered by coimmunoprecipitation (co-IP) (Fig. 7, *I* and *J*). In addition, consistent with this, we detected an interaction between PRMT1 and C/EBPβ in 3T3-L1 cells (Fig. 7, *K* and *L*). Since PRMT1 catalyzes arginine methylation of histone and nonhistone proteins, we suspected PRMT1 might affect the arginine methylation of C/EBPβ protein. To test this hypothesis, PRMT1 and C/EBPβ were cotransfected into 293T cells and then immunoprecipitation (IP) was adopted to measure the asymmetric methylation of C/EBPβ. Surprisingly, PRMT1 resulted in the decreased asymmetrically methylated C/EBPβ level in 293T cells (Fig. S6*A*), while PRMT1 deletion contributed to the raised asymmetrically methylated C/EBPβ level in 3T3-L1 cells (Fig. S6*B*). We then hypothesized that PRMT1 might affect the arginine methylation of C/EBPβ through other PRMT isoforms. Recent study has shown that depletion of PRMT1 in adipocytes leads to an increase in mRNA levels of type I PRMTs, including PRMT3, PRMT4, PRMT6, and PRMT8, in which the highest increase is PRMT6 (19). Besides, PRMT1 is reported to suppress PRMT6 in skeletal muscle and myoblasts, resulting in the depressed methylation of FOXO3 (27). Similarly, in our study, we also observed the upregulated expression of PRMT6 in PRMT1 knockdown 3T3-L1 cells (Fig. S6, *C* and *D*), indicating PRMT1 might influence methylation of C/EBPβ indirectly *via* PRMT6. Of note, phosphorylated C/EBPβ exhibited a significant decrease in PRMT1-deficient 3T3-L1 cells (Fig. 7*M*), which is considered as active C/EBPβ to trigger the transcription of PPARγ and C/EBPα (6). Taken together, our findings indicate that PRMT1 interacts with C/EBPβ and enhances the phosphorylation of C/EBPβ.

### PRMT1 acts as a positive regulator of CEBP/β protein stability by downregulating Smurf2

**Figure 8:**
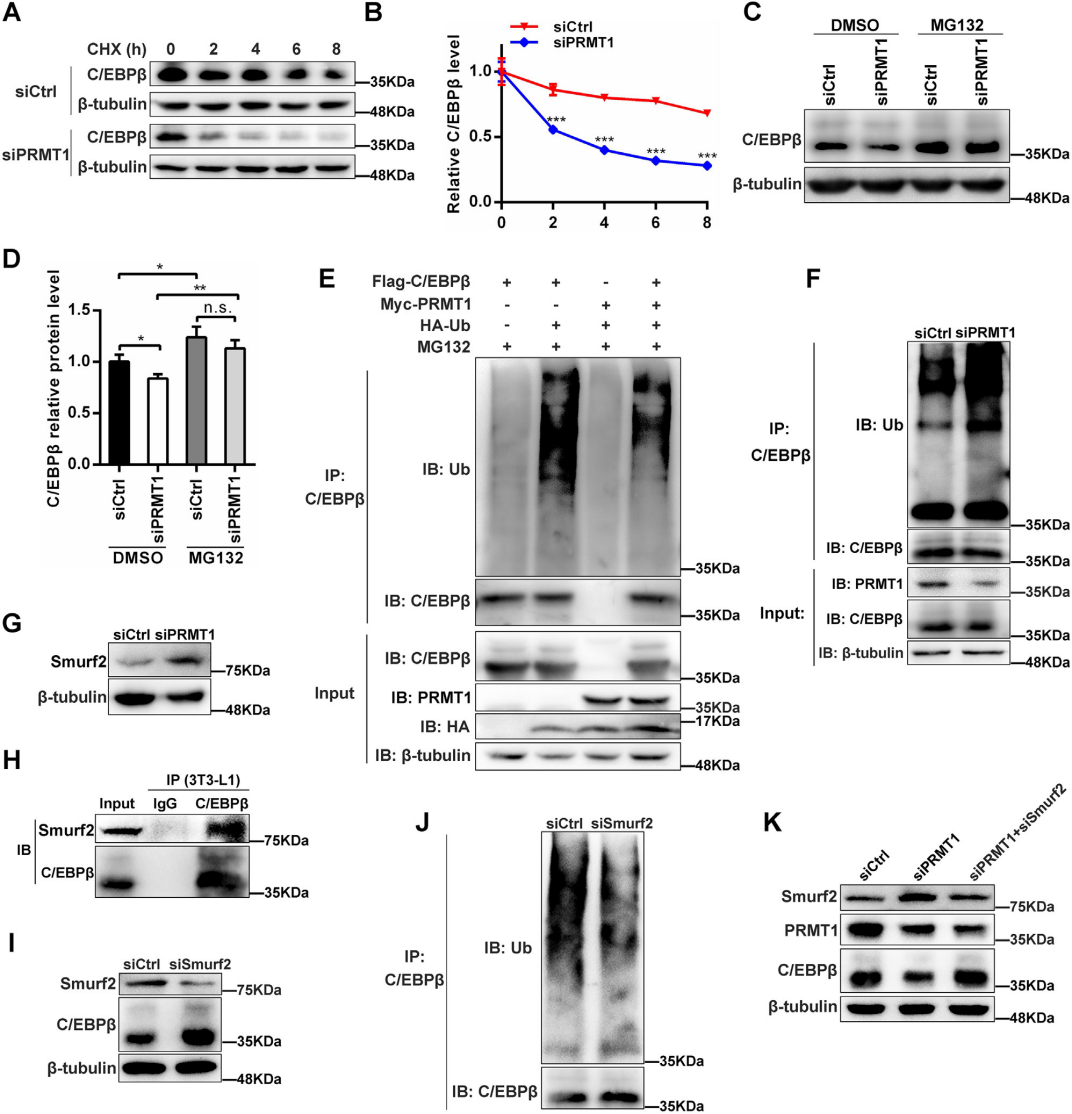
**PRMT1 promotes the protein stability of C/EBPβ by decreasing the level of Smurf2.***A*, siCtrl or siPRMT1 preadipocytes were incubated with DMI for 24 h and then treated with CHX. Cells were harvested at the indicated time points and analyzed by immunoblotting. *B*, quantification of the immunoblotting signals from (*A*). *C*, 3T3-L1 cells were transfected with siCtrl or siPRMT1 and induced for adipogenesis. After induction for 1 day, cells were treated with vehicle (DMSO) or MG132(10 μM) for 12 h and then analyzed by Western blot. *D*, quantification of the C/EBPβ protein levels relative to loading control β-tubulin. *E*, 293T cells were transfected with plasmids expressing Flag-C/EBPβ, Myc-PRMT1, and HA-Ub as indicated for 24 h and treated with MG132 for 12 h. Cells were lysed for co-IP with anti-C/EBPβ antibody and detected by immunoblotting with anti-Ub antibody. *F*, 3T3-L1 cells were transfected with siCtrl or siPRMT1 on confluence. After DMI induction for 24 h, cells were treated with MG132 for 12 h. The ubiquitination of C/EBPβ was determined by immunoprecipitation using anti-C/EBPβ antibody and immunoblotting with anti-Ub antibody. *G*, Western blot analysis of the protein expression of Smurf2. *H*, C/EBPβ interacts with Smurf2. 3T3-L1 cells with the treatment of DMI for 2 days were lysed for immunoprecipitation with anti-C/EBPβ antibody and then analyzed by Western blot as indicated. *I*, protein level of C/EBPβ and Smurf2 was detected when Smurf2 was silenced in 3T3-L1 cells with the treatment of DMI for 2 days. *J*, 3T3-L1 cells were transfected with siCtrl or siSmurf2 on confluence. After DMI induction for 24 h, cells were treated with MG132 for 12 h. The ubiquitination of C/EBPβ was determined by immunoprecipitation using anti-C/EBPβ antibody and immunoblotting with anti-Ub antibody. *K*, 3T3-L1 cells were transfected with siCtrl, siPRMT1, or siSmurf2 as indicated and the protein expression of Smurf2, PRMT1, and C/EBPβ were analyzed by Western blot after DMI induction for 2 days. CHX, cycloheximide; DMSO, dimethyl sulfoxide.

Our aforementioned data suggested that PRMT1 interacts with C/EBPβ and elevates its protein level. We next addressed how PRMT1 regulates the protein level of C/EBPβ. PRMT1 has been proven to be associated with protein stability and ubiquitination in multiple cells (28, 29, 30, 31). To pinpoint whether PRMT1 affects the stability of C/EBPβ, 3T3-L1 cells transfected with siCtrl or siPRMT1 were harvested after the translational inhibitor cycloheximide pretreatment for 0, 2, 4, 6, and 8 h and then C/EBPβ protein expression was analyzed. The protein level of C/EBPβ in control group decreased in a time-dependent manner (Fig. 8*A*). Compared to controls, C/EBPβ was more highly degraded and had a shorter half-life in PRMT1 knockdown 3T3-L1 cells (Fig. 8*B*), indicating PRMT1 might contribute to the stability of C/EBPβ. In addition, MG132 was employed to protect C/EBPβ protein from degradation mediated by ubiquitin-proteasome pathway in 3T3-L1 cells. As anticipated, MG132 treatment significantly increased the protein level of C/EBPβ in both siCtrl and siPRMT1 3T3-L1 cells and was able to effectively recue the attenuated protein level of C/EBPβ triggered by PRMT1 silencing (Fig. 8, *C* and *D*). Notably, PRMT1 reduced the ubiquitination of C/EBPβ in 293T cells (Fig. 8*E*). On the contrary, the ubiquitination of C/EBPβ was enhanced in PRMT1 knockdown 3T3-L1 cells (Fig. 8*F*).

**Figure 9:**
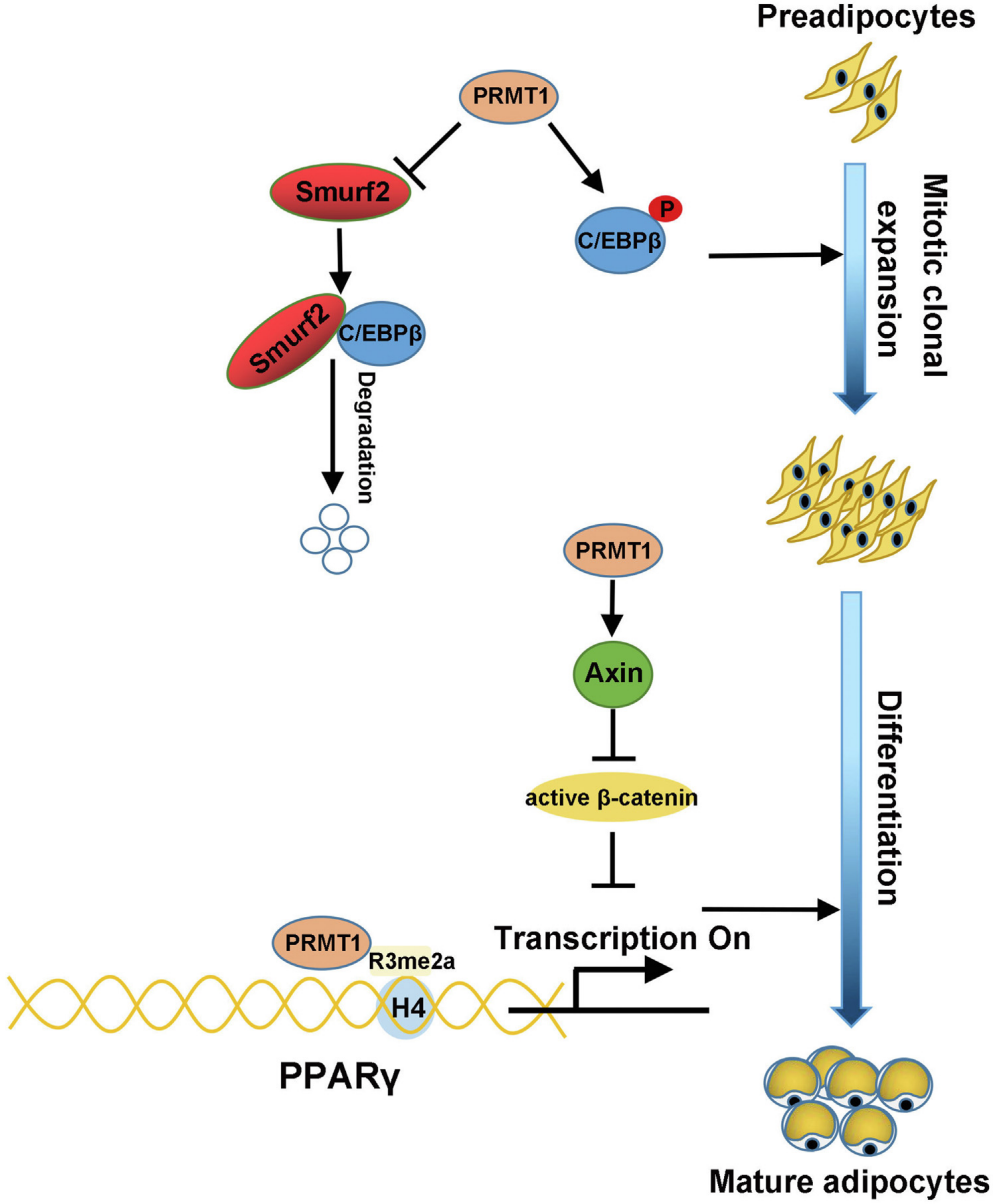
**Mechanism scheme for the role of PRMT1 in adipogenesis.** PRMT1 interacts with C/EBPβ and accelerates its phosphorylation to promote mitotic clonal expansion. Moreover, PRMT1 enhances the protein stability of C/EBPβ by reducing the level of E3 ubiquitin ligase Smurf2 that promotes the ubiquitination and degradation of C/EBPβ. Besides, PRMT1 regulates PPARγ expression by mediating H4R3me2a and reinforces its transactivity on target genes. Meanwhile, PRMT1 hinders the activation of Wnt/β-catenin signaling by increasing the level of Axin to promote adipocyte differentiation. Therefore, PRMT1 promotes adipogenesis through positively regulating C/EBPβ and PPARγ and negatively regulating Wnt/β-catenin signaling.

We struggled to probe into the mechanism by which PRMT1 promoted the protein stability of C/EBPβ. It was wondrously found that PRMT1 deletion in 3T3-L1 cells contributed to the increased protein expression of Smurf2 (Fig. 8*G*), a member of E3 ligase family. Similarly, research has shown that PRMT1 methylates Smurf2 and regulate its stability in HeLa cells (32). We wonder if PRMT1 might affect C/EBPβ stability by Smurf2. To validate our speculation, co-IP was employed to investigate the relationship between Smurf2 and C/EBPβ, confirming that C/EBPβ interacted with Smurf2 in 3T3-L1 cells (Fig. 8*H*). Furthermore, when Smurf2 was silenced in 3T3-L1 cells, the protein expression of C/EBPβ was notably augmented (Fig. 8*I*), while the ubiquitination of C/EBPβ was markedly diminished (Fig. 8*J*). Indeed, cotransfection with siPRMT1 and siSmurf2 in 3T3-L1 cells rescued the reduced protein of C/EBPβ resulted from PRMT1 deficiency (Fig. 8*K*). In sum, these results identify E3 ligase Smurf2 regulates the ubiquitylation and degradation of C/EBPβ in 3T3-L1 cells and uncover PRMT1 promotes C/EBPβ stability by negatively regulating Smurf2. We put forward a model to illustrate how PRMT1 regulates adipogenesis in Figure 9.

## Discussion

Recent studies of mouse models have shown that PRMT1 participates in metabolism in various tissues. Cardiac-specific PRMT1 ablation induces myocardium hypertrophy and heart attack through CaMKII dysregulation (33), while skeletal muscle–specific Prmt1 KO leads to muscle atrophy *via* hyperactivated FOXO3 with enhanced energy deprivation (27). Deletion of hepatic PRMT1 in hyperglycemic *db*/*db* mice causes the inhibition of FOXO1-dependent gluconeogenesis (34). Adipocyte-specific depletion of Prmt1 impairs glucose homeostasis by promoting the activation of the AMPK pathway, resulting in decreased fat mass and increased lipophagy (19). Likewise, we found PRMT1 is involved in metabolic process of adipocytes *in vitro*. The formation of new fat cells through adipogenesis responding to excess caloric energy is essential for metabolic health and the expansion of adipose depots (35, 36). Despite PRMT1 is of great significance to metabolism, its function and underlying mechanisms in adipogenesis remain unclear. In the present study, we reveal that PRMT1 regulates the expression of C/EBPβ and PPARγ at the posttranslational and transcriptional level, respectively, ultimately leading to the promotion of adipogenesis.

Adipogenesis is highly orchestrated by a complicated network of regulators, of which PPARγ is the master regulator. PPARγ inactivation causes severe lipodystrophy and insulin resistance, and precursor cells fail to induce any lipid accumulation when lack of PPARγ, that cannot be even restored by ectopic expression of C/EBPα (9, 10). We find that PRMT1 is required for adipocyte differentiation, lipid accumulation, and adipogenic genes expression, including PPARγ, in both 3T3-L1 cells and C3H10T1/2 cells, indicating a regulatory effect of PRMT1 on PPARγ expression. This finding is further testified by RNA-seq. Furthermore, PRMT1 deficiency leads to the decreased enrichment of H4R3me2a at PPARγ promoter. Consistent with our finding, PRMT1 has been demonstrated to regulate macrophage differentiation by catalyzing H4R3me2a methylation at the PPARγ promoter (24). Arg3 methylation on histone H4 by PRMT1 is closely related to transcriptional activation (23). Indeed, PPARγ overexpression rescues the inhibition of adipocyte differentiation and adipogenesis marker genes expression caused by PRMT1 knockdown. In addition, PRMT1 methyltransferase inhibitor TC-E 5003 significantly suppresses adipocyte differentiation and the expression of PPARγ and its target genes. To summarize, our data indicate that PRMT1 regulates adipogenesis in a PPARγ-dependent manner.

Many important signaling pathways orchestrate the process of preadipocyte differentiation into mature adipocytes, including Wnt/β-catenin signaling and Hedgehog signaling. It is widely spread that Wnt signaling plays a vital role in the differentiation of mesenchymal stem cells into mature adipocytes (6). Autocrine Wnt expression restrains terminal differentiation into mature adipocytes from precursors, while perturbation of Wnt signaling by Axin overexpression in preadipocytes promotes these cells to differentiate into adipocytes (37). Previous study has shown that PRMT1 directly interacts with and methylates Axin to enhance its stability (20). In this work, we find that knockdown of PRMT1 decreases the level of Axin and accelerates the level of active β-catenin. Conversely, overexpression of PRMT1 reduces the level of active β-catenin. Moreover, the inhibition of lipid accumulation triggered by PRMT1 depletion can be partially rescued by Wnt inhibitor IWR-1 endo that stabilizes Axin to block Wnt signaling pathway. Wnt/β-catenin signaling has been demonstrated to inhibit adipogenesis *via* reducing the expression of PPARγ and C/EBPα (21). Inhibiting Wnt/β-catenin signaling by IWR-1 endo restores the expression of PPARγ and C/EBPα in the absence of PRMT1. As a consequence, PRMT1 regulates adipogenesis through Wnt/β-catenin signaling.

MCE is a precondition for adipocyte terminal differentiation. In the early stage of adipogenesis, growth-retarded preadipocytes reentry into the cell cycle in response to adipogenic stimuli and go through MCE, followed by sequential expression of adipocyte phenotype-specific genes (7). Here, we show that PRMT1 deficiency inhibits MCE and leads to the downregulated expression of cyclin D1 and cyclin E1 and the upregulated expression of p53. p53 plays a vital role in adipocyte development, adipose tissue homeostasis, and metabolism (38). PRMT1 regulates splicing of Mdm4, controlling p53 levels *via* proteasomal degradation pathways in epicardial-derived cell lineages (26). Thus, it is worthwhile in further work to explore whether PRMT1 may regulate alternative splicing of Mdm4 to influence adipogenesis. C/EBPβ is crucial for MCE, and the blocked MCE is likely due to the lessened level of C/EBPβ. This suggestion is supported by the observation that C/EBPβ restores the attenuated proliferation by PRMT1 depletion during MCE. Although PRMT1 has little effect on the transcription of C/EBPβ, it is able to augment the protein level of C/EBPβ, which indicates the role of PRMT1 on C/EBPβ at posttranscriptional level. Interestingly, we find that PRMT1 directly interacts with C/EBPβ. PRMT1 is responsible for the majority of asymmetric dimethylation in mammalian cells (39). However, PRMT1 knockdown aggrandizes the methylation of C/EBPβ. In contrast, overexpression of PRMT1 leads to the lowered methylation level of C/EBPβ. Besides, PRMT1 depletion accelerates the expression of other type 1 PRMT family members, such as PRMT6 and PRMT4. Similarly, depletion of Prmt1 in both adipocytes and eWAT results in an increase of type I PRMTs, including PRMT6 (19). PRMT1 indirectly modulates arginine methylation of FOXO3 by repressing the expression of PRMT6 in skeletal muscle and eWAT (19, 27). Thus, PRMT1 may indirectly regulate C/EBPβ methylation *via* PRMT6 in adipocytes. It has been recently shown that the interaction of C/EBPβ with SWI/SNF and mediator complexes is restrained by arginine methylation mediated by PRMT4, and there is a negative regulation between phosphorylation and methylation of C/EBPβ (13). Adipogenic induction requires the phosphorylation and activation of C/EBPβ by MAP kinase and GSK3β (6). Contrary to the effect on methylation, PRMT1 positively regulates the phosphorylation of C/EBPβ. Previous studies have suggested that PRMT1 participates in regulating protein stability (28, 29, 30, 31). PRMT1 interacts not only with ribosome and proteasome constituents but also with deubiquitinases, such as USP7, that promotes protein stability (28). Asymmetrically dimethylated EZH2 by PRMT1 impedes the phosphorylation and ubiquitylation of EZH2 to regulate breast cancer metastasis (31). PRMT1 methylates RBM15 and leads to its degradation *via* ubiquitylation by E3 ligase CNOT4 (30). Indeed, our findings show that PRMT1 enhances the stability of C/EBPβ and prevents its ubiquitylation by downregulation of Smurf2 protein level. Smurf2 is a HECT-type E3 ubiquitin ligase as a negative regulator of TGF-β signaling (40). Smurf2 is methylated by PRMT1, and knockdown of PRMT1 resulted in the increased Smurf2 expression in HeLa cells (32). However, little is known about the roles of Smurf2 in adipogenesis or the relationship with C/EBPβ. We identify that it serves as E3 ligase to promote the ubiquitylation and degradation of C/EBPβ. Moreover, Smurf2 is also an E3 ubiquitin ligase for Axin that mediates proteasomal degradation of Axin, and knockdown of endogenous Smurf2 results in reduced β-catenin (41). Therefore, it is intriguing to deepen the understanding whether PRMT1 may regulate the protein level of Axin by Smurf2 in the future.

In conclusion, this study reveals that PRMT1 is essential for adipogenesis, orchestrating both MCE and terminal differentiation. The mechanisms underlying the effects of PRMT1 on adipogenesis involve positive regulation of C/EBPβ and PPARγ at the posttranslational and transcriptional level, respectively. PRMT1 reinforces C/EBPβ stability by downregulation of E3 ligase Smurf2 protein. H4R3me2a at PPARγ promoter mediated by PRMT1 triggers the transcription of PPARγ. In addition, PRMT1 negatively regulates the activation of Wnt/β-catenin signaling by upregulation of Axin protein level. These findings supply a new approach to delineate the molecular mechanism of adipogenesis and provide potential therapeutic targets for obesity.

## Experimental procedures

### Cell culture and differentiation

3T3-L1 and C3H10T1/2 cells (ATCC) were grown in Dulbecco’s modified Eagle’s medium (DMEM, Gibco) with 10% fetal bovine serum (FBS, Gibco) and 1% penicillin/streptomycin. For adipogenic differentiation of 3T3-L1 cells, cells were treated with 1 μM dexamethasone, 500 μM 3-isobutyl-1-methylxanthine, 10 μg/ml insulin (DMI) when cells reached confluence for 2 days. After 3 days, DMI medium was replaced by 10% FBS medium containing only 10 μg/ml insulin, and cells were maintained in this medium for 3 days. C3H10T1/2 cells were grown to 50% confluence and then committed into the adipogenic lineage by treating with 10 ng/ml BMP4 recombination protein, in which they were maintained until 2 days post-confluence. Then C3H10T1/2 cells were induced to adipocyte differentiation using the same aforementioned cocktail.

### siRNA and plasmids transfection

For PRMT1 knockdown, targeted siRNAs and nontargeted control siRNA were purchased from Invitrogen. The sequences of all siRNAs were listed in Table S1. The coding sequence of mouse PRMT1 was cloned into pcDNA3.1-Myc vector to generate pcDNA3.1-Myc-PRMT1. Plasmids C/EBPβ-Flag and HA-Ub were purchased from Miaolingbio. Lipofectamine 3000 transfection reagent (Invitrogen) was utilized for transfection with siRNAs or plasmids as per manufacturer’s instructions. The transfected cells were induced to differentiation after 2 days of confluence.

### PRMT1 inhibitor treatment

TC-E 5003 (APE×BIO), a selective PRMT1 inhibitor (42), was used to specifically investigate the roles of PRMT1 methyltransferase activity during adipogenic differentiation. Cells were cultured in adipogenic medium supplemented with TC-E 5003 or vehicle (dimethyl sulfoxide) on 2 days post-confluence. After 6 days of differentiation, ORO or Bodipy staining was employed to confirm the lipid droplet accumulation.

### Wnt signaling inhibitor treatment

Cells were treated with IWR-1 endo (APE×BIO), a specific β-catenin inhibitor stabilizing Axin, which is a scaffold protein of the β-catenin destruction complex to block Wnt signaling pathway (43). After transfection of siPRMT1 for 12 h，the culture medium was substituted by fresh DMEM containing 10% FBS and 5 μM IWR-1 endo until 2 days post-confluence. Then differentiation was induced by displacing the medium with DMEM combined with 10% FBS, DMI, and IWR-1 endo.

### RNA extraction and real-time quantitative PCR

Total RNA was extracted from cultured cells by TRIzol reagent (Magen) and reverse transcription was conducted by StarScript II First-strand cDNA Synthesis Kit (GenStar). Quantitation of the mRNA levels by qPCR was performed on a real-time PCR system LightCycler 480 (Roche) using SYBR Green Master Mix (GenStar). Relative mRNA levels were normalized by endogenous reference gene (β-actin). The primers used for qPCR are listed in Table S2.

### Western blot

Cells were lysed in radioimmunoprecipitation assay protein extraction buffer (FDbio) within 1% protease inhibitor cocktail. The lysed samples were centrifuged at 14, 000*g* and 4 °C for 10 min and supernatants containing protein were collected. The concentrations of proteins were quantified by bicinchoninic acid regent (Beyotime) according to the manufacturer’s protocol and proteins were boiled at 100 °C for 10 min. Equivalent amount of protein samples were separated in 10% SDS-PAGE and transferred to polyvinylidene difluoride membrane (Millipore). After blocking, membranes were incubated overnight at 4  °C with the specific primary antibodies shown in Table S3. The membranes were washed and then incubated with corresponding secondary antibodies for 1 h at room temperature (RT). Blots were visualized by the ECL chemiluminescence system.

### IP

Co-IP was performed using Dynabeads Protein G IP Kit (10007D, Invitrogen) following the IP protocol. Cells were lysed in IP lysis buffer for 30 min. Dynabeads were rotatably incubated with indicated antibodies for 2 h at RT. Cell lysates were collected for IP and incubated with indicated Dynabeads–antibodies complex overnight at 4 °C. The beads were washed four times in wash buffer. Then immunoprecipitates and input were applied for Western blot. The antibodies used for IP are listed as follows: anti-PRMT1 (2449s, Cell Signaling Technology), anti-Ubiquitin (3933s, Cell Signaling Technology), anti-C/EBPβ (ab32358, abcam), anti-Flag (8146s, Cell Signaling Technology), and anti-Myc (2276s, Cell Signaling Technology).

### Luciferase reporter assay

Luciferase reporter assays were implemented according to product instruction (Promega). CEBP/ɑ-promoter luciferase vector and renilla luciferase-expressing plasmid (pRL) were cotransfected with PRMT1 plasmids, PPARγ plasmids, or empty vector into 293T cells. After 36 h transfection, cells were lysed and enzymic reactions were assayed. The firefly luciferase activity was normalized to renilla luciferase internal control.

### ORO

Adipocytes were stained by ORO for determination of lipid accumulation. Cells were fixed with 4% formaldehyde for 10 min and washed by 60% isopropanol, followed by incubation with the working solution of ORO (Sigma–Aldrich; ORO:deionized water = 6:4) for 1 h at RT in the dark. After staining, cells were washed twice with distilled water and visualized using bright-field microscopy (Nikon). Finally, ORO was collected from the cells using 100% isopropanol, and absorbance was measured at 510 nm.

### Bodipy staining

Cells were fixed with 4% paraformaldehyde for 30 min and permeabilized with 0.1% of Triton X-100 in PBS for 20 min. After being washed with PBS for three times, cells were stained with Bodipy for 30 min, and the nucleus was stained with 4′,6-diamidino-2-phenylindole (DAPI) for 3 min. Photograph were captured by microscope (Nikon).

### Immunofluorescence and confocal microscopy

After DMI induction for 1 day, 3T3-L1 cells were fixed with 4% paraformaldehyde for 30 min and permeabilized with 0.1% of Triton X-100 in PBS for 20 min. Samples were then blocked in 4% bovine serum albumin in PBS for 1 h at RT and incubated with mouse anti-PRMT1 antibody (sc-166963, Santa Cruz) and rabbit anti-C/EBPβ antibody (ab32358, abcam) in 1% bovine serum albumin/PBS overnight at 4 °C. Washed three times with PBS, cells were then incubated with antimouse IgG secondary antibody–conjugated Alexa Fluor 488 and anti-rabbit IgG secondary antibody–conjugated Alexa Fluor 555 for 1 h at RT. Nuclei were stained by DAPI, and samples were analyzed by confocal microscopy (Leica).

### EdU assay

Cells were cultured in DMEM supplemented with 10 μM EdU for 1 h on 1 day post-confluence of 3T3-L1 cells. Then cells were fixed and EdU staining was conducted to check the proliferation of 3T3-L1 cells on MCE according to the supplier’s instructions (RiboBio), and nucleus was stained with DAPI.

### Cell viability assay

3T3-L1 cells viability was detected using CCK-8 kit (Yeasen) in accordance with the manufacturer’s protocol. In brief, cells were seeded on 96-well cell culture plates at a density of 5000 cells per well. Subsequently, 3T3-L1 cells were treated with DMI to induce adipogenic differentiation on confluence, and then, 10 μl CCK-8 reagent was added at 0, 24, and 48 h. After 0.5 h of incubation, the cell viability was computed by measuring the absorbancy at 450 nm.

### Chromatin immunoprecipitation assay

Chromatin immunoprecipitation assay was performed as described previously (44). Briefly, cells were crosslinked with 1% formaldehyde for 10 min at RT and quenched for 5 min by adding 0.125 M glycine. After harvested in SDS lysis buffer and sonication, DNA–protein complexes were immunoprecipitated with Chromatin immunoprecipitation-grade protein G magnetic beads (Cell Signaling Technology) and corresponding antibodies against H4R3me2a (39705, Active Motif), C/EBPβ (ab15050, abcam), or IgG (2729s, Cell Signaling Technology). After IP, DNA samples were analyzed by qPCR and normalized to IgG or inputs. The primers used are listed in Table S4.

### RNA-seq

RNA-seq was performed by Wuhan IGENEBOOK Biotechnology. Briefly, total RNA was purified from siCtrl and siPRMT1 3T3-L1 cells at DMI induction for 1 day and 3 days using the RNAprep Pure Kit DP432 (TIANGEN Biotech Co, Ltd) following protocol. The integrity of the total RNA was assessed by Qsep1 instrument. The libraries were prepared by MGIEasy mRNA Library Prep Kit, and 3 μg of total RNA was used as input. Indexed libraries were sequenced using MGI 2000. The raw reads were filtered out through cutadapt (version 1.11). Clean reads were mapped to the *Mus musculus* reference transcripts by Hisat2 (version 2.1.0) (45). Abundance of transcripts and normalization of expression was calculated as fragments per kilobase of transcript per million mapped reads. DESeq2 was applied to compute differentially expressed genes: adjusted q-value <0.05 (Benjamini–Hochberg corrected *p*-value) and |log2[fold change]| >0.5 (46). GO analysis was estimated using hypergeometric distribution with a q-value cutoff of 0.05.

### Statistical analysis

Statistical analyses were performed using unpaired two-tailed Student's *t* test by GraphPad Prism (GraphPad Software). Data were presented as mean ± SD from at least three independent experiments. *p* value <0.05 was regarded as significant difference.

## Data availability

The RNA-seq data are available online at NCBI GEO (GSE197065). All the rest of data are contained within the article and the supporting information.

## Supporting information

This article contains supporting information.

## Conflict of interest

The authors declare that they have no conflicts of interest with the contents of this article.
