# Supplementary material for: Protein arginine methyltransferase PRMT1 promotes adipogenesis by modulating transcription factors C/EBPβ and PPARγ
Source: J Biol Chem. 2022 Jul 31;298(9):102309. doi: 10.1016/j.jbc.2022.102309 (PMC9425039; doi:10.1016/j.jbc.2022.102309)
Supplement: Supporting information [file mmc1.docx]

**Protein arginine methyltransferase PRMT1 promotes adipogenesis by modulating transcription factors C/EBPβ and PPARγ**

Qi Zhu^†^, Dinghui Wang^†^, Feng Liang, Xian Tong, Ziyun Liang, Xiaoyu Wang, Yaosheng Chen, Delin Mo*

*From the State Key Laboratory of Biocontrol, School of Life Sciences, Sun Yat-sen University, Guangzhou, Guangdong, China*

† These authors contributed equally to this work.

* For correspondence: Delin Mo，modelin@mail.sysu.edu.cn

**Supplementary information**

**Page 2** Figure S1

**Page 3** Figure S2

**Page 4** Figure S3, Figure S4

**Page 5** Figure S5, Figure S6

**Page 6** Table S1, Table S2, Table S3

**Page 7** Table S4


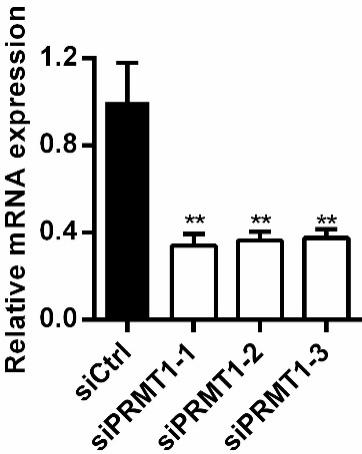


**Figure S1. Knockdown efficiency determination of siRNAs against PRMT1.** 3T3-L1 cells were transiently transfected with siCtrl, siPRMT1-1, siPRMT1-2 or siPRMT1-3 on confluence, respectively. The efficiency of siRNAs to silence PRMT1 in 3T3-L1 cells was determined by qRT-PCR after transfection for 48 h. Data are represented as mean ± SD (n = 3). Student’s t-test, ****P*<0.001.


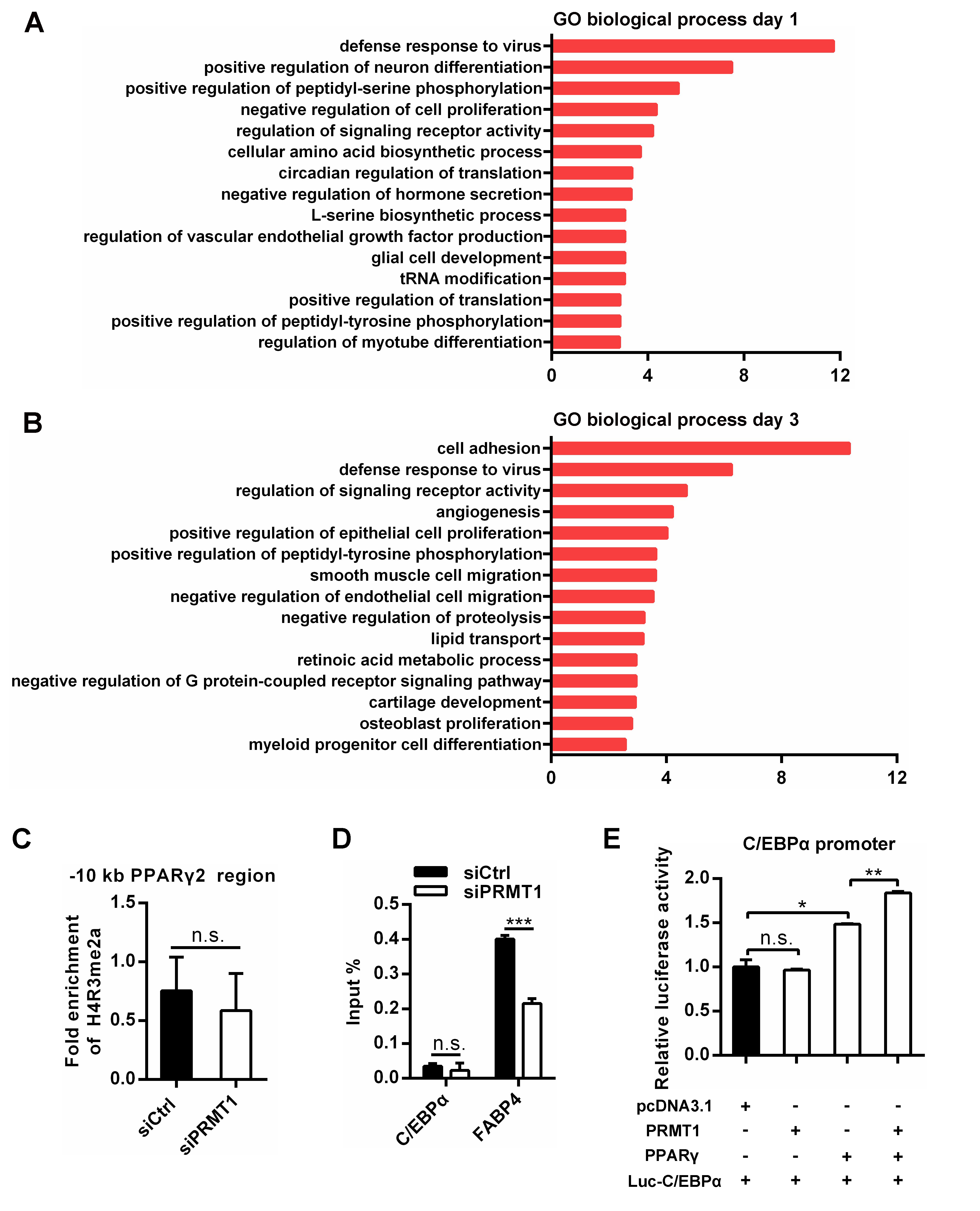


**Figure S2. PRMT1 regulates adipogenesis by epigenetic modification.** *A,* Gene ontology (GO) analysis of up-regulated genes at day 1 by RNA seq. The *P* value for the enrichment of biological process GO-term is shown. *B,* GO analysis of up-regulated genes at day 3 involved in cell adhesion, lipid transport and other biological process. *C*, PRMT1 expression in 3T3-L1 cells was silenced. Then the binding of H4R3me2a to the sequences approximately 10 kb upstream of PPARγ2 promoter was detected by ChIP-qPCR in adipocytes. IgG was used as a negative control. Data are represented as mean ± SD (n = 3). Student’s t-test, n.s. not significance. *D*, ChIP-qPCR analysis the enrichment of PRMT1 in C/EBPɑ and FABP4 promoter. Data are presented as mean ± SD (n = 3). ****P*<0.001, n.s. not significant (Student’s t-test). *E*, 293T cells were co-transfected with empty vector, PRMT1 and PPARγ plasmids as indicated, together with luciferase reporter plasmid containing C/EBPɑ promoter and renilla internal control reporter plasmid. Luciferase activity was measured after transfection for 36 h. Data are presented as mean ± SD (n = 3). **P*<0.05, ***P*<0.01, n.s. not significant (Student’s t-test).


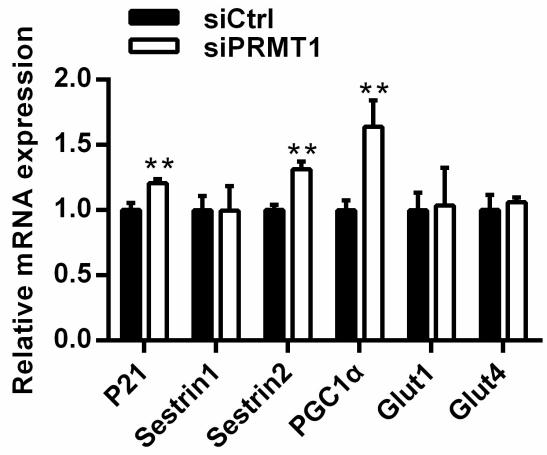


**Figure S3. The mRNA expression of p53 target genes.** 3T3-L1 cells were transfected with siCtrl and siPRMT1 and qRT-PCR analyzed the expression of p53 target genes on DMI 2 days.


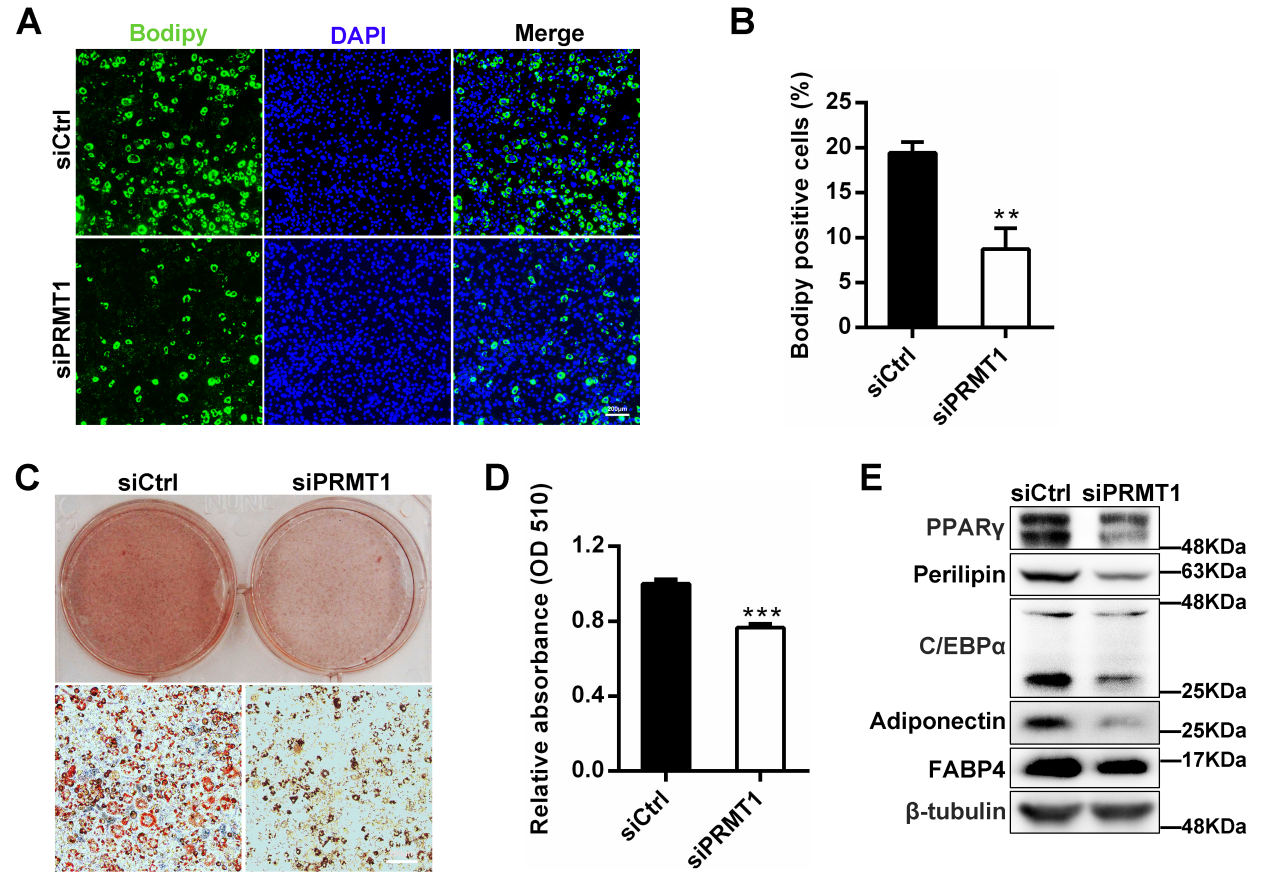


**Figure S4. PRMT1 can regulate adipogenic differentiation independent of MCE.** *A*, 3T3-L1 cells were transfected with siCtrl and siPRMT1 after MCE. Lipid accumulation was assessed by Bodipy staining at six days after differentiation induction. Scale bar represents 200 µm. *B*, the percentage of Bodipy positive cells was counted as shown in A. Data are represented as mean ± SD (n = 3). ***P*<0.01. *C*, 3T3-L1 cells were transfected with siCtrl and siPRMT1 after MCE. Adipogenic differentiation was evaluated by oil red O staining at six days after differentiation induction. Scale bar represents 200 µm. *D*, the OD values were measured at 510 nm as shown in C. Results are the mean ± SD (n = 3). Student’s t-test, ****P*<0.001. *E*, 3T3-L1 cells were transfected with siCtrl and siPRMT1 after MCE. Western blot analysis was performed to detect the protein levels of adipogenic marker genes.


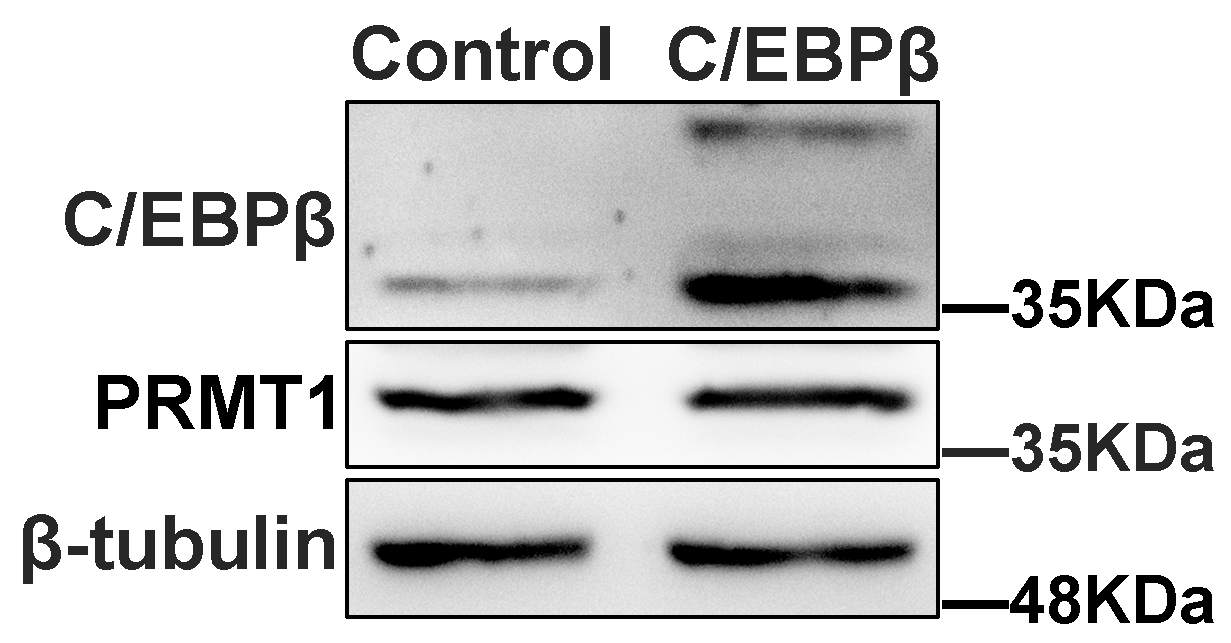


**Figure S5. C/EBPβ does not affect the expression of PRMT1.** Control or C/EBPβ plasmids were transfected into 3T3-L1 cells on confluence. Western blot analyzed the level of C/EBPβ and PRMT1 during MCE.


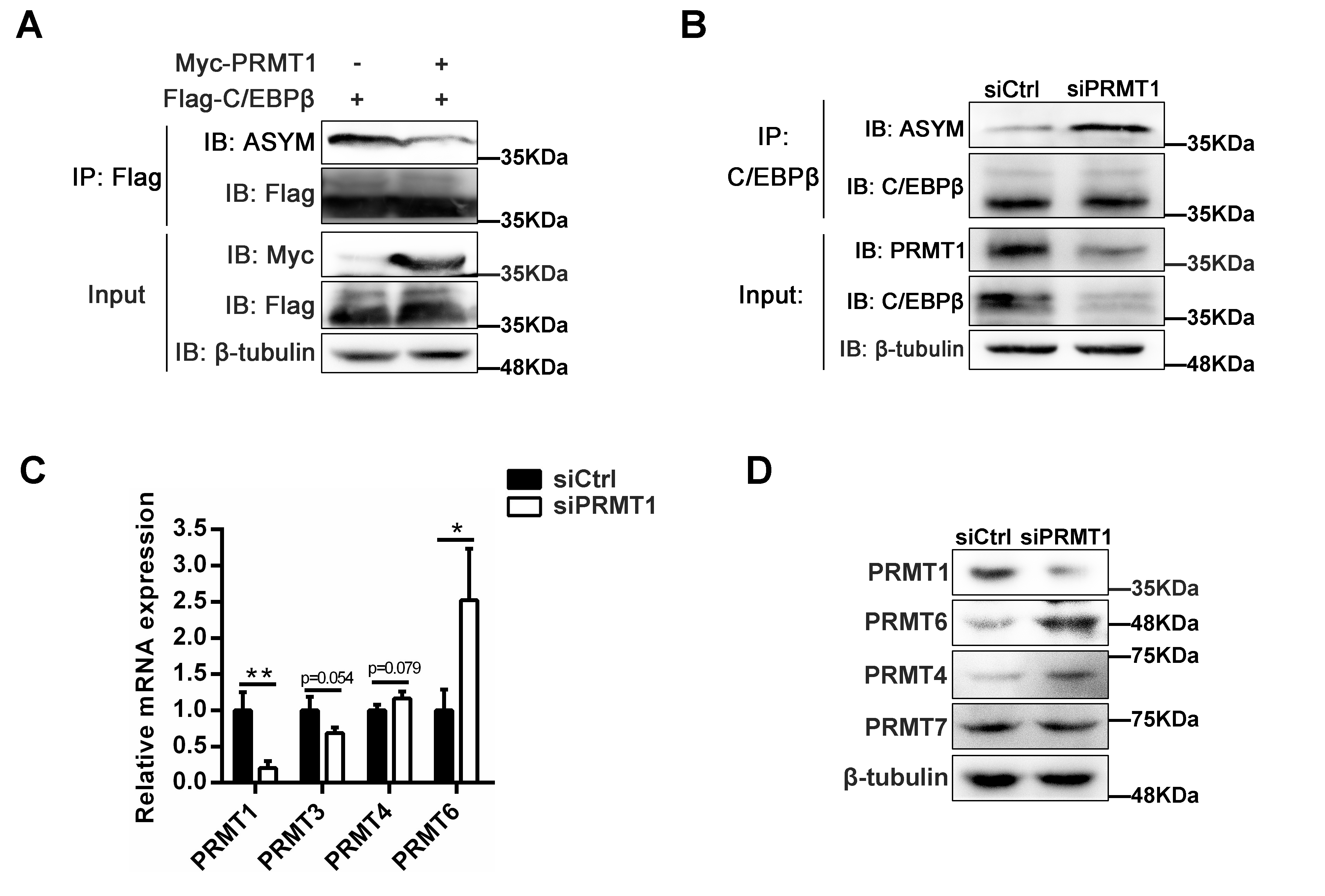


**Figure S6. PRMT1 indirectly affects the arginine methylation of C/EBPβ.** *A,* 293T cells were cotransfected with Myc-PRMT1 and Flag-C/EBPβ plasmids. The methylation of C/EBPβ was determined by immunoprecipitation using anti-Flag antibody and immunoblotting with anti-ASYM antibody. *B,* co-IP was performed to observe the asymmetric arginine dimethylation of C/EBPβ in siCtrl and siPRMT1 3T3-L1 cells. *C,* qRT-PCR analysis of Type I PRMTs levels from control and PRMT1 knockdown 3T3-L1 cells. Data are mean ± SD (n = 3). Student’s t-test, **P*<0.05, ***P*<0.01. *D,* immunoblot analysis for PRMT1, PRMT4, PRMT6 and PRMT7 protein expression in control and PRMT1 knockdown 3T3-L1 cells.

| **Table S1 siRNA sequence** | |
| --- | --- |
| siRNA | Sequence |
| siPRMT1-1(MSS205235) | CCAUGUUUCACAAUCGGCAUCUCUU |
|  | AAGAGAUGCCGAUUGUGAAACAUGG |
| siPRMT1-2(MSS205236) | CCAUUGGAAUGAGGCCCAAUGCCAA |
|  | UUGGCAUUGGGCCUCAUUCCAAUGG |
| siPRMT1-3(MSS274953) | CAGCCAUUGAGGACCGACAAUAUAA |
|  | UUAUAUUGUCGGUCCUCAAUGGCUG |
| Smurf2 | CCGUCUUCCUGAUCCAUUUTT |
|  | AAAUGGAUCAGGAAGACGGTT |

| **Table S2 The primers for qRT-PCR** | | |
| --- | --- | --- |
| Gene | Sequence | |
| PRMT1 | F:CCTCACATACCGCAACTCCA | R:CATCCAGCACCACCTTGTCT |
| PPARγ | F:TGCTGTTATGGGTGAAACTCT | R:CGCTTGATGTCAAAGGAATGC |
| CEBP/ɑ | F:GGCTCTCATTCTTTTTGGTTTAGGG | R:CTAAGACCCACTACTACATACACCC |
| FABP4 | F:AAGTGGGAGTGGGCTTTGC | R:CCGGATGGTGACCAAATCC |
| CEBP/β | F:TGGACAAGCTGAGCGACGAG | R:TGTGCTGCGTCTCCAGGTTG |
| β-actin | F:GTGCTATGTTGCTCTAGACTTCG | R:ATGCCACAGGATTCCATACC |
| P21 | F: CCTGGTGATGTCCGACCTG | R:CCATGAGCGCATCGCAATC |
| Glut4 | F:TCTTGGACGGTTCCTCATTG | R:GGCGATTTCTCCCACATACA |
| Glut1 | F:GCTGTGCTTATGGGCTTCTC | R:AGAGGCCACAAGTCTGCATT |
| Sesn1 | F:GTCTGGATAACATCACATTAG | R:CCAGGTAGGAACACTGATGC |
| Sesn2 | F:TAGCCTGCAGCCTCACCTAT | R:TATCTGATGCCAAAGACGCA |

| **Table S3 List of antibodies** | | | | | |
| --- | --- | --- | --- | --- | --- |
| Antibody | Application | | Catalog number | Vendor | |
| PRMT1 | WB | | ab7027 | Abcam | |
| PPARγ | WB | | 2435s | Cell Signaling Technology | |
| CEBP/α | WB | | 2295s | Cell Signaling Technology | |
| β-tubulin | WB | | 2146s | Cell Signaling Technology | |
| FABP4 | WB | | 2120s | Cell Signaling Technology | |
| H4R3me2a | WB, Chip | | 39705 | Active Motif | |
| β-catenin | WB | | 9562s | Cell Signaling Technology | |
| active-β-catenin | WB | | 8814s | Cell Signaling Technology | |
| ASYM25 | WB | | 09-814 | Millipore | |
| H3 | WB | | ab201456 | Abcam | |
| P57 | WB | | ab75974 | Abcam | |
| CyclinA2 | WB | | ab137769 | Abcam | |
| CyclinE1 | WB | | 20808s | Cell Signaling Technology | |
| P53 | WB | | sc-126 | Santa Cruz | |
| CyclinD1 | WB | | 2978s | Cell Signaling Technology | |
| CEBP/β | WB, IF, IP | | ab32358 | Abcam | |
| p-CEBP/β | WB | | 3084T | Cell Signaling Technology | |
| CEBP/β | Chip | | ab15050 | Abcam | |
| PRMT1 | IF | | sc-166963 | Santa Cruz | |
| PRMT1 | IP | | 2449s | Cell Signaling Technology | |
| Ubiquitin | IP | | 3933s | Cell Signaling Technology | |
| Flag | IP | | 8146s | Cell Signaling Technology | |
| Myc | IP | | 2276s | Cell Signaling Technology | |
| PRMT1 | Chip | | ab190892 | Abcam | |
| H3K27me3 | Chip | | 9733s | Cell Signaling Technology | |
| H3K4me3 | Chip | | 9751s | Cell Signaling Technology | |
| Adiponectin | WB | | 2789s | Cell Signaling Technology | |
| Axin | WB | | 2087s | Cell Signaling Technology | |
| Smurf2 | WB,IP | | 12024s | Cell Signaling Technology | |
| PRMT6 | WB | | sc-271744 | Santa Cruz | |
| PRMT7 | WB | | sc-376077 | Santa Cruz | |
| PRMT4 | WB | | sc-390656 | Santa Cruz | |
| **Table S4 The primers for ChIP-qPCR** | | | | | |
| Gene | | Sequence | | | |
| PPARγ promoter | | F:TTCAGATGTGTGATTAGGAG | | | R:AGACTTGGTACATTACAAGG |
| PPARγ2 promoter | | F:ACAGTTCACACCCCTCACAA | | | R:TGGCACTGTCCTGATTGAGA |
| PPARγ2 -10 kb upstream region | | F:GGGCGTTAAAACACAATCCT | | | R:TCTCTTCCTCCTTCCCTTCC |
| CEBP/α promoter | | F:TCCCTAGTGTTGGCTGGAAG | | | R:CAGTAGGATGGTGCCTGCTG |
| FABP4 promoter | | F:GAGCCATGCGGATTCTTG | | | R:CCAGGAGCGGCTTGATTGTTA |
